# FFAR2 expressing myeloid-derived suppressor cells drive cancer immunoevasion

**DOI:** 10.1186/s13045-024-01529-6

**Published:** 2024-02-24

**Authors:** Zeda Zhao, Juliang Qin, Ying Qian, Chenshen Huang, Xiaohong Liu, Ning Wang, Liqin Li, Yuqing Chao, Binghe Tan, Na Zhang, Min Qian, Dali Li, Mingyao Liu, Bing Du

**Affiliations:** 1 Shanghai Frontiers Science Center of Genome Editing and Cell Therapy, Shanghai Key Laboratory of Regulatory Biology and School of Life Sciences, Institute of Biomedical Sciences and School of Life Sciences, East China Normal University, 500 Dongchuan Road, Shanghai, 200241 China; 2 Fujian Provincial Hospital, Fuzhou, China; 3 Huzhou Central Hospital, Affiliated Hospital of Zhejiang University, Zhejiang, China; 4 BRL Medicine Inc., Shanghai, China

**Keywords:** SCFAs, FFAR2, MDSCs, Immune evasion, Immunotherapy

## Abstract

**Background:**

Emerging evidences suggest that aberrant metabolites contributes to the immunosuppressive microenvironment that leads to cancer immune evasion. Among tumor immunosuppressive cells, myeloid-derived suppressor cells (MDSCs) are pathologically activated and extremely immunosuppressive, which are closely associated with poor clinical outcomes of cancer patients. However, the correlation between MDSCs mediated immunosuppression and particular cancer metabolism remained elusive.

**Methods:**

Spontaneous lung adenocarcinoma and subcutaneous mouse tumor models, gas chromatography–mass spectrometry (GC–MS) and immunofluorescence assay of patient-derived lung adenocarcinoma tissues, and flow cytometry, RNA sequencing and Western blotting of immune cells, were utilized.

**Results:**

Metabolite profiling revealed a significant accumulation of acetic acids in tumor tissues from both patients and mouse model, which contribute to immune suppression and cancer progression significantly through free fatty acid receptor 2 (FFAR2). Furthermore, FFAR2 is highly expressed in the myeloid-derived suppressor cells (MDSCs) from the tumor of lung adenocarcinoma (LUAD) patients which is greatly associated with poor prognosis. Surprisingly, whole or myeloid *Ffar2* gene deletion markedly inhibited urethane-induced lung carcinogenesis and syngeneic tumor growth with reduced MDSCs and increased CD8^+^ T cell infiltration. Mechanistically, FFAR2 deficiency in MDSCs significantly reduced the expression of Arg1 through Gαq/Calcium/PPAR-γ axis, which eliminated T cell dysfunction through relieving L-Arginine consumption in tumor microenvironment. Therefore, replenishment of L-Arginine or inhibition to PPAR-γ restored acetic acids/FFAR2 mediated suppression to T cells significantly. Finally, FFAR2 inhibition overcame resistance to immune checkpoint blockade through enhancing the recruitment and cytotoxicity of tumor-infiltrating T cells.

**Conclusion:**

Altogether, our results demonstrate that the acetic acids/FFAR2 axis enhances MDSCs mediated immunosuppression through Gαq/calcium/PPAR-γ/Arg1 signaling pathway, thus contributing to cancer progression. Therefore, FFAR2 may serve as a potential new target to eliminate pathologically activated MDSCs and reverse immunosuppressive tumor microenvironment, which has great potential in improving clinical outcomes of cancer immunotherapy.

**Supplementary Information:**

The online version contains supplementary material available at 10.1186/s13045-024-01529-6.

## Background

Hematopoietic stem cells (HSCs)-derived tumor-infiltrating myeloid cells constitute the most abundant heterogeneous immune-related cells in the tumor microenvironment, and play essential roles in tumor immune evasion [1–3]. Among them, myeloid-derived suppressor cells (MDSCs) are pathologically activated and extremely immunosuppressive, which are closely associated with poor clinical outcomes of cancer patients [4]. Emerging studies demonstrated that MDSCs contribute to the myeloid cell diversity in pathological conditions. Whereas, the nature of this diversity and the characteristics for the distinction of MDSCs from neutrophils and monocytes have been poorly understood [5]. Generally, MDSCs can be divided into granulocytic/polymorphonuclear MDSCs (PMN-MDSCs) and monocytic MDSCs (M-MDSCs) according to the strong phenotypic and morphological distinction. Actually, the complex pathological environment leads to specific genomic, proteomic and metabolic features of MDSCs which enable their particular immune suppression for tumor evasion. Thus, characterizing and exploring the features and function of different substantial proportion of MDSCs are essential for targeting myeloid cells to improve current immunotherapeutic regimens or to overcome resistance to immunotherapy.

The initiation and progression of tumors depend on the metabolic reprogramming of cancer cells to fulfill their bioenergetic and biosynthetic demands to support rapid proliferation as well as constitute immune suppressive tumor microenvironment (TME) [6]. Not only tumor cell-derived cytokines, but also low pH and hypoxia environment [7] are all involved in regulation of MDSCs mediated immune suppression [8]. Moreover, enhanced free fatty acids (FFAs) uptake, fatty acid oxidation (FAO) and lipids present are all benefit MDSCs mediated immunosuppression obviously [9]. In spite of the dominant role of lactic acid in TME, short-chain fatty acids (SCFAs) including acetate (C2), propionate (C3) and butyrate (C4) have long been considered as key signaling molecules in numerous physiological and pathological processes [10]. However, previous studies mainly focus on the regulation of host homeostasis by intestinal microbiota produced SCFAs. Recent studies suggested the existence of a de novo pathway for acetate production derived from pyruvate, the end product of glycolysis [11]. Hyperactive metabolism such as Warburg effect in tumors leads to increased glucose uptake, incomplete metabolism, and the release of metabolic intermediates, like acetate, into the extracellular space [12]. Whereas, the immune regulation of SCFAs in tumor microenvironment is rarely explored.

The accumulation of SCFAs in tumor microenvironment not only enhanced the SCFAs uptake and carbon metabolism of immune cells, but also increased the G protein signaling through free fatty acid receptors (FFARs) which belong to the family of G protein-coupled receptors (GPCRs). As a classic GPCR, FFAR2 couples to Gαi/o and Gαq, resulting in inhibition of the adenylate cyclase pathway or increasing intracellular calcium levels [13, 14]. Although, FFAR2 has been found to play a critical role in migration of polymorphonuclear leukocytes (PMNs) [15] and regulate the size and function of Treg pool through gut microbiota-derived SCFAs [16], the function of FFAR2 in MDSCs mediated tumor immune evasion remains unclear.

Here, we report a significant accumulation of SCFAs (acetic acids) in both patients and mouse tumor tissues, and FFAR2 activated by acetic acids in a more immune suppressive MDSCs, which are proven essential for immune suppression and cancer progression. Furthermore, we also demonstrated the non-redundant role of FFAR2 in MDSCs mediated L-Arginine consumption through Gαq/Calcium/PPAR-γ/Arg1 signaling. Moreover, pharmaceutical inhibition of FFAR2 signaling significantly facilitated the immune checkpoint blockade (ICB) mediated tumor suppression, suggesting the great potential of FFAR2 as a novel target for cancer immune therapy.

## Materials and methods

### Cell preparation and culture

Mouse Lewis lung carcinoma cell (LLC), melanoma cell (B16F10), fibroblast cell (3T3), human melanoma cell (SK-MEL-2), lung adenocarcinoma cell (A549), human normal epithelial cell (BEAS-2B) and umbilical vein endothelial cell (HUVEC) lines were purchased from the American Type Culture Collection (ATCC, USA). NCM460 and PC-9 cell line was obtained from Cell Resource Center of East China Normal University. LLC was cultured in Dulbecco’s Modified Eagle’s Medium (DMEM) supplemented with 10% fetal bovine serum (FBS) and 1 × penicillin–streptomycin. LLC stably expressing firefly luciferase (LLC-luci) was generated by our lab as described [17, 18] and cultured in complete DMEM medium with 200 ng/mL G418 (Gibco). B16F10, HUVEC, PC-9, A549 and 3T3 were cultured in RPMI 1640 Medium supplemented with 10% fetal bovine serum (FBS), 1 × penicillin–streptomycin. SK-MEL-2 was cultured in Modified Eagle’s Medium (MEM) supplemented with 10% fetal bovine serum (FBS), 1 × penicillin–streptomycin. All cell lines cultured maintained at 37 °C and 5% CO_2_, and were regularly tested for mycoplasma-free.

### Chemicals, reagents, and antibodies

RPMI 1640, Dulbecco’s modified Eagle’s medium (DMEM), penicillin–streptomycin, and fetal bovine serum (FBS) was purchased from Gibco. TRIzol reagent and PrimeScript RT Master Mix were acquired from Takara. SYBR Green PCR Master Mix was purchased from Yeasen. Mouse TNF-α ELISA kit was purchased from Biolegend and Mouse IL-12 p70 ELISA kit was purchased from Invitrogen. GM-CSF and IL-6 were purchased from Proteintech. InVivoMab anti-mouse Ly6G/Ly6C (Gr-1; clone RB6-8C5), InVivoMab IgG2a isotype antibody (clone LTF-2) and InVivoMab anti-mouse PD-1 (clone RMP1-14) were purchased from BioXCell. Mouse Myeloid-Derived Suppressor Cell Isolation Kit, mouse CD4^+^ T Cell Isolation Kit, mouse CD8^+^ T Cell Isolation Kit, LS Separation columns, MS Separation columns, MACS BSA Stock Solution, autoMACS Rinsing Solution, autoMACS Running Buffer and mouse T Cell Activation/Expansion Kit were purchased from Miltenyi. FFAR2 Agonist (#371725), FFAR2 inhibitor (GLPG0974, #SML2443), Sodium acetate (#S2889) and Urethane (#U2500) were purchased from Sigma-Aldrich. Gαq inhibitor (YM-254890) and FFAR2 inhibitor (CATPB) were purchased from MCE. Ca^2+^ inhibitor (2-APB) was purchased from Tocris and PPAR-γ-inhibitor (GW9662) was purchased from Selleck. Live/dead dye and antibodies used for flow cytometry were purchased from Biolegend unless indicated otherwise: Fixable Viability Dye (BD, Horizon™ Fixable Viability Stain 780), FITC-conjugated anti-mouse CD45 (clone 30-F11), APC-conjugated anti-mouse CD3 (clone 17A2), Brilliant Violet 421™-conjugated anti-mouse CD4 (clone RM4-4), PE-conjugated anti-mouse CD8a (clone 53-6.7), PE/Cyanine7-conjugated anti-mouse F4/80 (clone BM8), PE-conjugated anti-mouse CD11c (clone N418), TruStain fcX™ anti-mouse CD16/32 (clone 93), Brilliant Violet 605™-conjugated anti-mouse I-A/I-E (clone M5/114.15.2), PE-conjugated anti-mouse CD8a (BD Pharmingen, clone 53-6.7), BV421-conjugated anti-mouse LY-6G (BD Pharmingen, clone 1A8), APC-conjugated anti-mouse LY-6C (BD Pharmingen, clone AL-21), FITC-conjugated anti-mouse CD11b (BD Pharmingen, clone M1/70), FITC-conjugated anti-mouse CD8a (clone 53-6.7), PE-conjugated anti-human/mouse Arginase 1 (eBioscience™, clone A1exF5), Brilliant Violet 421™-conjugated anti-mouse/human CD11b (clone M1/70), PE-conjugated anti-mouse IFNγ (clone XMG1.2), Brilliant Violet 421™-conjugated anti-mouse/human CD11b (clone M1/70), PE-conjugated anti-mouse IFNγ (clone XMG1.2), FITC-conjugated anti-mouse CD8a (clone 53-6.7), PE-conjugated anti-mouse iNOS (clone W16030C), PE/Cyanine7-conjugated anti-mouse IL-10 (clone JES5-16E3), Alexa Fluor® 700 -conjugated anti-mouse NK-1.1 (clone PK136) and Brilliant Violet 421™-conjugated anti-mouse CD25 (clone A18246A). Antibodies used for Western blotting are as follows: PPAR-γ (#2443), Arginase-1 (#93668), STAT1 (#9172), p-STAT1 (#9171), STAT3 (#4904), p-STAT3 (#9145) and AlexaFluor® 488/555 mouse, rabbit secondary antibodies were purchased from Cell Signaling Technology (CST). C/EBPβ (H7, sc-7962) and p-C/EBPβ (Thr217, sc-16993-R) were purchased from Santa Cruz Biotechnology. Antibodies used for immunofluorescence are as follows: Anti-human FFAR2 (ab124272) and anti-human CD15 (ab17080) were purchased from abcam. Anti-mouse Gr-1 (clone RB6-8C5) was purchased from Biolegend. Anti-CD8 (GB13429), anti-CD4 (GB13064-2), anti-human ARG1 (GB11285) and anti-PPAR-γ (GB112205) were purchased from Servicebio.

### Mice

C57BL/6 mice (6–8 weeks old) were purchased from Gempharmatech Co., Ltd. *Lyz2-cre* mice were purchased from Jackson Laboratory. Global knockout mice *Ffar2*^*−/−*^ and conditional knockout mice *Ffar2*^*fl/fl*^ were constructed using the CRISPR–Cas9 genome-editing system. *Ffar2*^*fl/fl*^ mice were crossed with *Lyz2-cre* mice to obtain mice with targeted *Ffar2* deletion. All mouse breeding and mouse animal experiments are done at the specific-pathogen-free conditions Experimental Animal Center of East China Normal University. All animal experiments were approved by the Institutional Animal Ethics Committee of East China Normal University. The protocol was approved by the East China Normal University Center for Animal Research (m20230201).

### Short chain fatty acids (SCFAs) measured by GC–MS

Sample preparation of cell supernatants: 3T3, LLC, B16F10, BEAS-2B, NCM460, HUVEC, PC-9, A549 and SK-MEL-2 cells were cultured in recommended complete medium for a few days, and seeded then in 6-well plates (1 × 10^6^ cells per well), and cultured in RPMI 1640 supplemented with 10% FBS and 1 × penicillin–streptomycin (3 ml complete medium per well) for 16 h. Cell culture supernatants were then collected and kept in −80 °C. Preparation of urethane-induced lung cancer tissue: Urethane-induced mice lung cancer tissue and normal mice lung tissue was dissected, washed twice with precooled PBS, and kept then in −80 °C. All samples were sent to Suzhou Meixin Bioscience Co., Ltd and analyzed and quantified by GC–MS.

### Development of mouse MDSCs from bone marrow (BM) precursors

Bone marrow cells were harvested from tibias and femurs of C57BL/6 mice (6–8 weeks old), after red cell lysis, cell suspension was cultured RPMI 1640 supplemented with 1 × Penicillin and Streptomycin, 10% FBS, GM-CSF (40 ng/ml) and IL6 (40 ng/ml) medium for 4 days.

### Preparation of LLC tumor explant supernatants

Mice were subcutaneously injected with LLC (1 × 10^6^ cells/mouse), and LLC tumors were excised at day 21. LLC tumors were minced into small pieces (less than 3 mm in diameter) and re-suspended into T75 culture flask with RPMI 1640 medium (without FBS) and incubated for 16–18 h. After incubation, supernatants were collected. After centrifugation and filtration (0.22 μm filters), supernatants were directly used or kept in −80 °C.

### CD4^+^ T cells, CD8^+^ T cells and MDSCs isolation from mouse spleen

Disrupt spleen in recommended buffer and pass through 70 μm nylon cell strainer (Corning) and followed red blood cell lysis. Prepared single cell suspensions were determined cell number, and used for following cell isolation. CD4^+^ T cells and CD8^+^ T cells were isolated from naïve C57BL/6 mouse spleen via Mouse CD4^+^ T Cell Isolation Kit (Miltenyi) or Mouse CD8^+^ T Cell Isolation Kit (Miltenyi), following the manufacturer’s instructions. MDSCs were isolated from LLC tumor-bearing C57BL/6 mouse spleen via Myeloid-Derived Suppressor Cell Isolation Kit (Miltenyi), following the manufacturer’s instructions.

### CD8^+^ T cells isolation from LLC tumors of Ffar2^fl/fl^ and Ffar2^fl/fl^Lyz2-cre mouse

LLC cells were subcutaneously injected into *Ffar2*^*fl/fl*^ and *Ffar2*^*fl/fl*^*Lyz2-cre* mice (1 × 10^6^ cells/mouse), and LLC tumors were excised at day 21. LLC tumors were minced into small pieces (less than 3 mm in diameter) and resuspended with RPMI 1640, 400 U/mL Collagenase IV (Gibco) and 30 U/mL DNase I (Gibco). Tumor small pieces were incubated at 37 °C for half an hour. Stopping tumor samples digestion used complete medium (RPMI 1640 + 10% FBS), and tumor samples were filtered through 70 μm nylon cell strainer (Corning). After red cell lysis, and CD8^+^ T cells isolated from generated single-cell suspensions via Mouse CD8a Positive Selection Kit II (STEMCELL Technologies) following the manufacturer's instructions.

### Flow cytometry analysis

For tumor infiltrating immune leukocytes, tumor single-cell suspensions created as described (CD8^+^ T cells isolation from LLC tumors). For spleen infiltrating immune leukocytes, disrupt spleen in PBS supplemented with 2% FBS and pass through a 70 μm nylon cell strainer (Corning) and followed red blood cell lysis. All samples were blocked FcγII/III with anti-CD16/32 (BD Pharmingen) at 4 °C for 30 min, and surface marker was stained at 4 °C for 30 min. Samples were then stained with indicated fluorescence-conjugated antibodies. Fixable Viability Stain 780 (BD Pharmingen) was used to gate out non-viable cells. For intracellular Arg1 and IFNγ staining, Cytofix/Cytoperm Soln Kit (BD Pharmingen) was used to fix and permeabilize cells, following the manufacturer’s instructions. All samples were run on LSRFortessa (BD Pharmingen) and analyzed by FlowJo software (Tree Star).

### T cell suppression assays

CD4^+^ T cells, CD8^+^ T cells isolation from naïve mice spleen and MDSCs isolation from tumor-bearing mice spleen described as above. MDSCs were plated in the 48-well plates and cocultured with 1 μM carboxyfluorescein succinimidyl ester (CFSE) labeled CD4^+^ or CD8^+^ T cells at different ratios in the complete medium (RPMI 1640 supplemented with 10% FBS). For T cells activation, T Cell Activation/Expansion beads (Miltenyi) was added to coculture of T cells and MDSCs. After 72 h, T cells proliferation and IFNγ expression were measured by flow cytometry.

### Mouse tumor models

For urethane-induced lung cancer, *Ffar2*^+*/*+^, *Ffar2*^*−/−*^, *Ffar2*^*fl/fl*^ and *Ffar2*^*fl/fl*^*Lyz2-cre* mice were intraperitoneally injected with urethane (1 g/kg body weight in 200 μl PBS) once per week for 10 weeks, lung tissues were excised and collected at 28 weeks. Then, the lungs were soaked in 4% paraformaldehyde in a fixed shape for 2 weeks. After that, lung nodules were quantified and photographed. For mice subcutaneous tumor model, LLC, LLC-luci or B16F10 cells were injected subcutaneously into *Ffar2*^+*/*+^, *Ffar2*^*−/−*^, *Ffar2*^*fl/fl*^ and *Ffar2*^*fl/fl*^*Lyz2-cre* mice (1 × 10^6^ cells/mouse), and tumor volume assessed using calipers and calculated using the formula [(small diameter)^2^ × (large diameter) × 0.5]. For MDSCs deletion in vivo, *Ffar2*^+*/*+^ or *Ffar2*^*−/−*^ mice were injected subcutaneously with LLC cells (1 × 10^6^ cells/mouse) and injected intraperitoneally with isotype or anti-Gr-1 antibody (200 μg/mouse, every 4 days) from day 4 to day 23. For bone marrow chimeras, bone-marrow cells after red blood cell lysis were collected from *Ffar2*^+*/*+^ or *Ffar2*^*−/−*^ mice. Prepared *Ffar2*^+*/*+^ or *Ffar2*^*−/−*^ mice were lethally irradiated with 8.5 Gy, and lethally irradiated mice received bone marrow transplants from *Ffar2*^+*/*+^ or *Ffar2*^*−/−*^ mice. Ten weeks after transplantation, chimeric mice were subcutaneously injected with LLC (1 × 10^6^ cells/mouse), and tumor growth was recorded. For the combined treatment with FFAR2 inhibitor and anti-PD-1 antibody, WT mice were injected subcutaneously with LLC (1 × 10^6^ cells/mouse). LLC-tumor bearing mice treated with FFAR2 inhibitor (5 mg/kg per day, single esophageal gavage), anti-PD1 antibody (200 μg/mouse every 4 days), FFAR2 inhibitor + anti-PD1 antibody or control (PBS containing 0.5% DMSO). Tumor-bearing mice were treated starting at day 4 post-tumor injection. Tumor growth and survival curve were recorded in two independent experiments. For survival analysis, mice were euthanized when total tumor burden approached IACUC guidelines with a tumor burden exceeding 1500 mm^3^ in volume.

### Coinjection of MDSCs and mouse tumor cells

MDSCs were isolated from tumor-bearing mice spleen via Myeloid-Derived Suppressor Cell Isolation Kit (Miltenyi) as described. WT mice were then injected with tumor cells (LLC or B16F10; 5 × 10^5^ cells/mouse) or co-injected with tumor cells and* Ffar2*^+*/*+^ MDSCs (5 × 10^5^:5 × 10^5^) or tumor cells and *Ffar2*^*−/−*^ MDSCs (5 × 10^5^:5 × 10^5^). Tumor volumes were recorded.

### Histology and immunofluorescence assay

Urethane-induced lung cancer tissues, LLC and B16F10 tumors were dissected, and washed twice by precooled PBS. Samples were fixed then in 4% paraformaldehyde for overnight, and embedded into paraffin. All paraffin embedded samples were sent to Servicebio. Hematoxylin and eosin (H&E) and immunofluorescence assay were stained and analyzed by Servicebio.

### Western blotting analysis

Generated bone marrow-derived MDSCs were first resting overnight, and then stimulated by GM-CSF (40 ng/ml) + IL6 (40 ng/ml) for the indicated time. After stimulation, BM-MDSCs were harvested and lysed with radio immunoprecipitation assay (RIPA) buffer (CoWin Biosciences, China, catalog# CW2333) supplemented with complete Mini Protease and Phosphatase inhibitor Cocktail (Roche, catalog# 4693159001 and 4906837001). Cell lysates were separated by standard SDS-PAGE and analyzed by immunoblotting.

### Enzyme-linked immunosorbent assay (ELISA)

Sample preparation of cell supernatants: generated bone marrow-derived MDSCs were first resting overnight, and then stimulated by GM-CSF (40 ng/ml) + IL6 (40 ng/ml) for 48 h. Remove particulates by centrifugation and assay immediately or store samples at -80℃. Preparation of LLC tumor tissue extracts: Mice were subcutaneously injected with LLC (1 × 10^6^ cells/mouse), and LLC tumors were excised at day 21. Add appropriate amount of PBS to the tissue and mash it. Centrifuge at 3000 rpm for 10 min to take the supernatants and kept then in -80℃. The concentration of L-Arginine from cell supernatants and LLC tumor tissue extracts were quantified using mouse L-Arginine (L-Arg) ELISA Kit (Shanghai Coibo Bio Technology Co.Ltd). Consumption of L-Arginine by MDSCs was calculated according to the formula: (concentration of L-Arginine in medium-concentration of L-Arginine in culture supernatants) × volume. TNF-α and IL-12 p70 in the culture supernatants and lysates of MDSCs were determined by ELISA following the manufacturer's instructions.

### Patients

Human lung adenocarcinoma tissues and adjacent normal tissues were obtained from Huzhou Central Hospital, Affiliated Hospital of Zhejiang University (Huzhou, 313000, Zhejiang, China) and statements that informed written consent of lung adenocarcinoma patients were obtained. All tissues were collected and handled according to the ethical and safety procedures approved by the Clinical Ethics Committee of the Huzhou Central Hospital, Affiliated Hospital of Zhejiang University (reference ethics number 20180701-02). The lung cancer tissue array (HLugA180Su08) was purchased from Shanghai Outdo Biotech.

### RNA sequencing and analysis

*Ffar2*^+*/*+^ and *Ffar2*^*−/−*^ bone marrow-derived MDSCs (1 × 10^6^ cells/well) were seeded in a 6-well plate and cultured in complete RPMI 1640 medium overnight. After overnight resting, BM-MDSCs were re-stimulated by combination GM-CSF (40 ng/ml) with IL-6 (40 ng/ml) for 24 h. After that, RNA was extracted using the RNA extraction kit (Magen, R4801-02) and sequenced by BGI (Beijing Genomic Institute in ShenZhen). The sequencing data were filtered with SOAPnuke (v1.5.2) by removing reads containing sequencing adapters, removing reads whose low-quality base ratio (base quality less than or equal to 5) was more than 20%, and removing reads whose unknown base (“N” base) ratio was more than 5%. After this, the clean reads were obtained and stored in a FASTQ format. The clean reads were aligned to the reference genome using HISAT2 (v2.0.4). Fusion genes and differential splicing genes (DSGs) were detected through Ericscript (v0.5.5), and rMATS (v3.2.5). Bowtie2 (v2.2.5) was used to align the clean reads to the gene set, a database for this organism was built by BGI (Beijing Genomic Institute in ShenZhen), coding transcripts were included, and the expression levels of genes were calculated using RSEM (v1.2.12). Differential expression analysis was performed using DESeq2 (v1.4.5) with a *P* value ≤ 0.05 and |log_2_ (fold change)|≥ 0.5. Volcano map was plotted by using the EnhancedVolcano package in R (v4.5.0). GSEA of KEGG pathway was performed by clusterProfiler package and visualized by ggplot2 package and gseaplot2 package. *P* value ≤ 0.05 was set as the cut-off criteria.

### RNA extraction and quantitative real-time RT-PCR

Total RNA of cells or tissues was isolated with TRIzol (TaKaRa), and RNA concentration was measured by NanoDrop 2000 (Thermo Fisher Scientific). RNA was reverse transcribed into cDNA by PrimeScript RT Master Mix (TaKaRa). Quantitative real-time RT-PCR (RT-qPCR) was performed using the QuantStudio 3 Real Time PCR System (Applied Biosystems). The expression of each gene was normalized to the expression level of GAPDH and reported as relative mRNA expression (2^−ΔΔCt^) or fold change. The sequence-specific primers are shown in Additional file 2: Table S1.

### Statistical analysis

Statistical analyses were analyzed by Prism 6.0 (GraphPad Software). Statistical differences between two groups were analyzed using unpaired two-tailed Student’s *t*-test. The statistical differences for more than two groups were analyzed using ANOVA. Survival analysis was performed using the Log-rank (Mantel-Cox) test. All data were shown as mean ± SEM. *P* value ≤ 0.05 was considered to be statistically significant. (*P < 0.05, **P < 0.01, ***P < 0.001, ****P < 0.0001, NS, not significant).

## Results

### Aberrant metabolism of SCFAs in tumor microenvironment

**Fig. 1:**
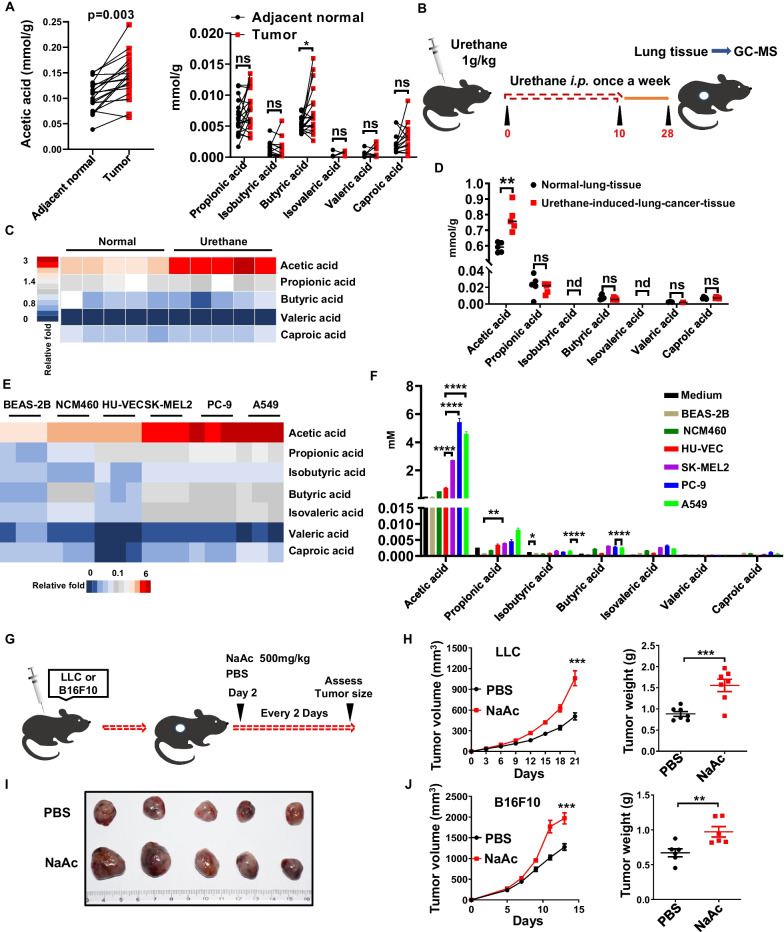
Tumor-derived acetic acids contribute to cancer progression. **A** Quantification of short chain fatty acids (SCFAs) in human adjacent normal tissues and human lung adenocarcinoma tissues measured by GC–MS (n = 21, biological replicates). **B**–**D** C57BL/6 mice were intraperitoneally injected with either urethane (1 g/kg body weight in 200 μl PBS, n = 5, biological replicates) or normal PBS (control, n = 5, biological replicates) once a week for 10 weeks, lung tissues were collected at 28 weeks for the quantification of short-chain fatty acids (SCFAs) via GC–MS (**B**). **C** and **D** Heat maps (**C**), and quantification (**D**) of SCFAs in lung tissue extracts of control (n = 5, biological replicates) and urethane-induced lung cancer mice (n = 5, biological replicates). **E** and **F** Heat maps (**E**), and quantification (**F**) of SCFAs in supernatants from 16 h cultures of human normal or tumor cells (1 × 10^6^ cells/well, n = 3, biological replicates). **G**–**J** LLC or B16F10 cells were injected subcutaneously into C57BL/6 mice (1 × 10^6^ cells/mouse, n = 6–7, biological replicates) and received intraperitoneal (*i.p.*) injections of PBS or NaAc (500 mg/kg) every two days from the second day (**G**). LLC tumor growth and tumor weight were recorded (**H**), and LLC tumor was photographed at the end of experiment (**I**). B16F10 tumor growth and tumor weight were recorded (**J**). **D**, **F**, **H** and **J** Data are shown as mean ± SEM, and the experiment was performed three times and a representative example is shown. **A**, **D**, **H** and **J** were analyzed by unpaired Student's *t*-test and **F** was analyzed by one-way ANOVA (*P < 0.05, **P < 0.01, ***P < 0.001, ****P < 0.0001 and NS, not significant)

To assess the aberrant SCFAs accumulation in tumor microenvironment, we employed gas chromatography-mass spectrometry (GC–MS)-based targeted metabolomics. Then acetic acids were predominantly accumulated in lung adenocarcinoma patients -derived tumor tissues (Fig. 1A). Similarly, acetic acids were also significantly increased in urethane-induced mice lung cancer tissues (Fig. 1B–D). Accordingly, both human (SK-MEL-2, PC-9 and A549) and mouse (B16F10 and LLC) tumor cells produced much more SCFAs (especially acetic acids) than control human HUVEC, BEAS-2B and NCM460 or mouse 3T3 cells (Fig. 1E, F, and Additional file 1: Fig. S1A and B). However, the serum acetic acids were little changed in tumor-bearing mice (Additional file 1: Fig. S1C), suggesting the specific accumulation of acetic acids in tumor microenvironment. Accordingly, elevated expression of ACLY and FASN (enzymes involved in fatty acid synthesis) was associated with reduced OS (overall survival) (Additional file 1: Fig. S1D and E). On the contrary, the enzymes (ACSS1 and ACSS2) involved in fatty acids degradation are extremely associated with increased OS (Additional file 1: Fig. S1F and G). Furthermore, intraperitoneal (*i.p.*) injections of NaAc (alternative mimetics of acetic acids) significantly increased tumor volume and weight in both LLC (Fig. 1G–I) and B16F10 (Fig. 1J) tumor model. These results suggest that metabolic reprogramming of cancer cells results in the accumulation of acetic acids in tumor microenvironment which facilitate the tumor progression.

### Tumor-derived acetic acids promote tumor progression through FFAR2

**Fig. 2:**
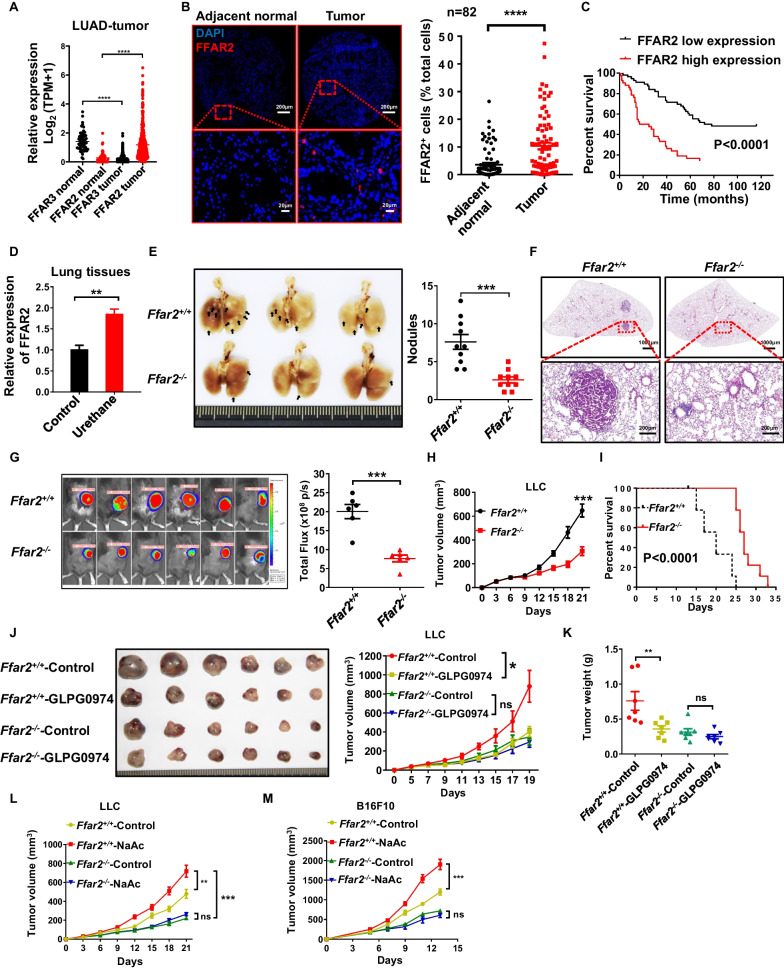
FFAR2 deletion suppress the formation and progression of lung cancer. **A** The expression of FFAR2 and FFAR3 in LUAD tumor tissue and normal tissue was obtained from TCGA-LUAD database. **B** and **C** Representative immunofluorescence staining images of FFAR2 (red) and quantification of FFAR2 positive cells (**B**) in lung adenocarcinoma tissues and adjacent normal tissues of human lung cancer tissue array (n = 82, biological replicates). **C** Log-rank (Mantel-Cox) test of cumulative survival rates of lung adenocarcinoma patients subdivided by FFAR2 high expression (with > 10% cells positive for FFAR2, n = 42, biological replicates) and FFAR2 low expression (with ≤ 10% cells positive for FFAR2, n = 56, biological replicates) in tumor tissues. **D** FFAR2 expression in control lung tissue and urethane induced lung cancer tissue were determined by real-time RT-qPCR (n = 3, biological replicates). **E** and **F** Urethane-induced lung tumor nodules in mice were photographed and quantified (**E**) (n = 10, biological replicates). **F** Representative histopathology of tumor lungs was analyzed by H&E staining (n = 5, biological replicates). **G** In vivo imaging of tumor mice (n = 6, biological replicates) was conducted following intraperitoneal administration of substrate (D-luciferin, 150 mg/kg). Representative bioluminescence images were shown, and total flux of fluorescence was quantified (**G**). **H** and **I** Tumor volume (**H**) (n = 6, biological replicates) and survival rate of mice bearing LLC tumors were recorded (**I**) (n = 9, biological replicates). **J** and **K** Mice bearing LLC tumors were treated with PBS containing 0.5% DMSO or FFAR2 inhibitor (GLPG0974, 5 mg/kg per day). Tumor growth (**J**) and tumor weight (**K**) were recorded. **L** and **M** *Ffar2*^+*/*+^ and *Ffar2*^*−/−*^ mice bearing LLC tumors (n = 6–8 mice/group) or B16F10 tumors (1 × 10^6^ cells/mouse, n = 6–8 mice/group) were intraperitoneally injected with PBS or NaAc (500 mg/kg). LLC tumor growth (**L**) and B16F10 (**M**) tumor growth were recorded. **D**–**M** Data are shown as mean ± SEM, and the experiment was performed three times and a representative example is shown. **D**–**H** were analyzed by unpaired Student's *t*-test and **J**–**M** were analyzed by two-way ANOVA (*P < 0.05, **P < 0.01, ***P < 0.001, ****P < 0.0001 and NS, not significant)

Although both FFAR2 and FFAR3 are receptors for SCFAs, only FFAR2 is highly expressed in tumor tissues (Fig. 2A, B) and positively correlated with poor prognosis of lung adenocarcinoma patients (Fig. 2C). In addition, the mRNA expression of FFAR2 was greatly increased in urethane-induced mice lung cancer tissues as compared to normal lung tissues (Fig. 2D). Furthermore, the number and size of urethane-induced tumor nodules (Fig. 2E), as well as solid adenoma lesions (Fig. 2F) were all significantly reduced in *Ffar2*^−/−^ mice. In addition, FFAR2 deficiency markedly restrained tumor growth (Fig. 2G, H) and prolonged the survival of syngeneic LLC (Fig. 2I) mouse model. Moreover, esophageal gavage of FFAR2 inhibitor GLPG0974 [19, 20] and intraperitoneal injection of another FFAR2 inhibitor CATPB significantly restrained the growth of LLC tumors in *Ffar2*^+/+^ mice, but not *Ffar2*^−/−^ mice (Fig. 2J, K, and Additional file 1: Fig. S2A). Whereas, the tumor growth of LLC (Fig. 2L) and B16F10 (Fig. 2M) could only be notably accelerated by NaAc in *Ffar2*^+/+^ mice. Meanwhile, NaAc and FFAR2 inhibitors could not influence the proliferation and survival of LLC and B16F10 tumor cells in vitro (Additional file 1: Fig. S2B–E). Collectively, these data demonstrate the predominant role of FFAR2 in acetic acids promoted tumor progression.

### Highly expression of FFAR2 contributes to the accumulation of MDSCs in tumor microenvironment

**Fig. 3:**
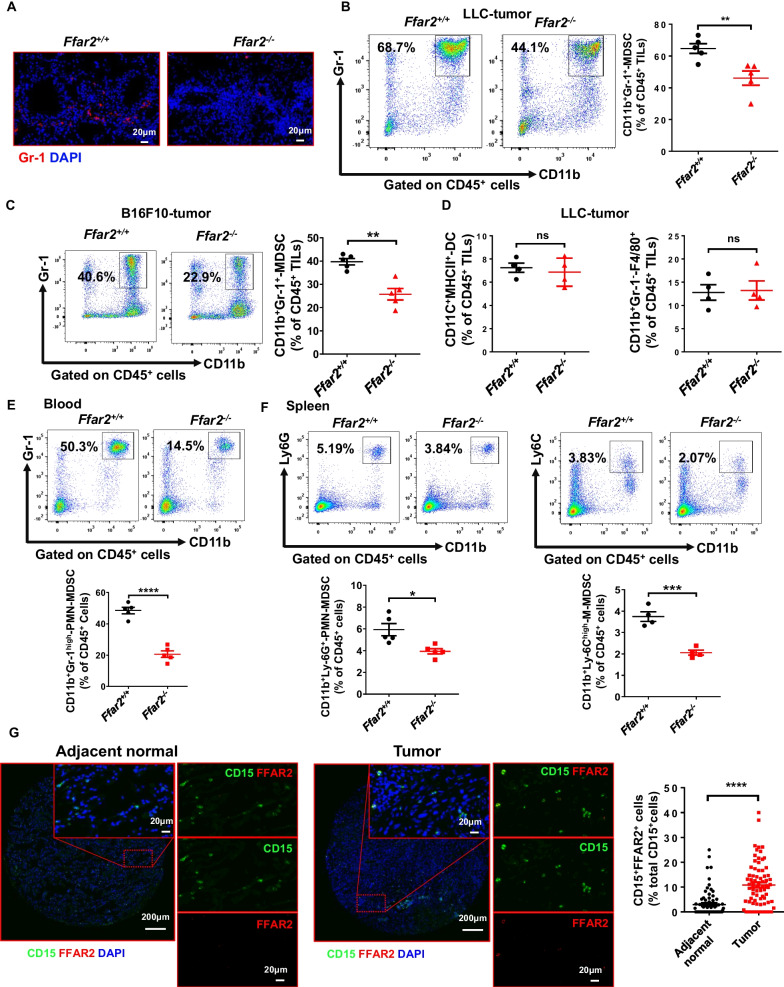
FFAR2 deletion reduces the accumulation of MDSCs. **A** Immunofluorescence analysis of infiltrating Gr-1^+^ cell in urethane-induced lung tumor nodules in mice (n = 5, biological replicates). **B** *Ffar2*^+*/*+^ and *Ffar2*^*−/−*^ mice were injected subcutaneously with LLC cells (1 × 10^6^ cells/mouse). After 21 d of transplantation, single cell suspensions were prepared from tumors and subjected to flow cytometry analysis (n = 5, biological replicates). The percentage of tumor-infiltrating CD11b^+^Gr-1^+^-MDSC of total CD45^+^ tumor-infiltrating leukocytes (TILs) in LLC tumors were shown. **C** *Ffar2*^+*/*+^ and *Ffar2*^*−/−*^ mice were injected subcutaneously with B16F10 cells (1 × 10^6^ cells/mouse). After 14 d of transplantation, single cell suspensions were prepared from tumors and subjected to flow cytometry analysis (n = 5, biological replicates). The percentage of tumor-infiltrating CD11b^+^Gr-1^+^-MDSC of total CD45^+^ tumor-infiltrating leukocytes (TILs) in B16F10 tumors in mice are shown. **D** Quantification of percentage of CD11C^+^MHCII^+^-DC and CD11b^+^Gr-1^−^-F4/80^+^-macrophage of total CD45^+^ tumor-infiltrating leukocytes (TILs) in LLC tumors (n = 4, biological replicates). **E** Mice were injected subcutaneously with LLC cells (1 × 10^6^ cells/mouse). After 21 d of transplantation, representative gating strategy and the percentage of CD11b^+^Gr-1^high^-PMN-MDSCs of total CD45^+^ cells in LLC-tumor bearing mouse blood were determined by flow cytometry (n = 5, biological replicates). **F** The percentage of CD11b^+^Ly-6G^+^-PMN-MDSC (n = 5, biological replicates) and CD11b^+^Ly-6C^high^-M-MDSC (n = 4, biological replicates) of total CD45^+^ cells in LLC-tumor bearing mouse spleen were determined by flow cytometry. **G** Representative images of immunofluorescence staining for CD15 and FFAR2 of human lung adenocarcinoma tissues with adjacent normal tissues, and the percentage of CD15^+^FFAR2^+^ cells in CD15^+^ total positive cells of human lung adenocarcinoma tissues and adjacent normal tissues (n = 73, biological replicates). **B**–**G** Data are shown as mean ± SEM, and the experiment was performed three times and a representative example is shown. Data were determined by unpaired Student's *t*-test (*P < 0.05, **P < 0.01, ***P < 0.001, ****P < 0.0001 and NS, not significant)

Tumor-infiltrating myeloid cells (TIMs) including MDSCs, TAMs and tumor-associated DCs are the most abundant immune-related cells in the TME, and TIMs play a key role in modulation of immunosuppressive tumor microenvironment [21]. Therefore, we investigated whether the effects of FFAR2 on tumor growth were through MDSCs, TAMs and DCs (Additional file 1: Fig. S3A). As shown in Fig. 3A–C, FFAR2 deficiency significantly attenuated Gr-1^+^ MDSC infiltration in urethane-induced mice lung tumor tissues, LLC and B16F10 tumor tissues. However, the other TIMs including CD11C^+^MHCII^+^-DCs and CD11b^+^Gr-1^−^F4/80^+^-TAMs were little changed (Fig. 3D and Additional file 1: Fig. S4A). Furthermore, CD11b^+^Gr-1^high^-PMN-MDSCs, CD11b^+^Ly-6G^+^-PMN-MDSCs and CD11b^+^Ly-6C^high^-M-MDSCs in the blood and spleens of *Ffar2*^−/−^ LLC tumor-bearing mice were also decreased significantly (Fig. 3E, F). And FFAR2 is also highly expressed in human lung adenocarcinoma tumor tissues (Fig. 3G) and tumor-bearing mice splenic MDSCs (Additional file 1: Fig. S4B). Both flow cytometry and immunofluorescent staining showed that the infiltration of CD4^+^ and CD8^+^ T cells was increased significantly in *Ffar2*^*−/−*^ LLC, B16F10 tumors and urethane-induced lung tumors (Additional file 1: Fig. S4C–E), while the infiltration of NK and Tregs was little changed (Additional file 1: Fig. S4F and G). Taken together, FFAR2 deletion reduces MDSCs accumulation, but increases CD4^+^ and CD8^+^ T cell infiltration in tumors significantly.

### FFAR2 deficient MDSCs suppress tumor growth

**Fig. 4:**
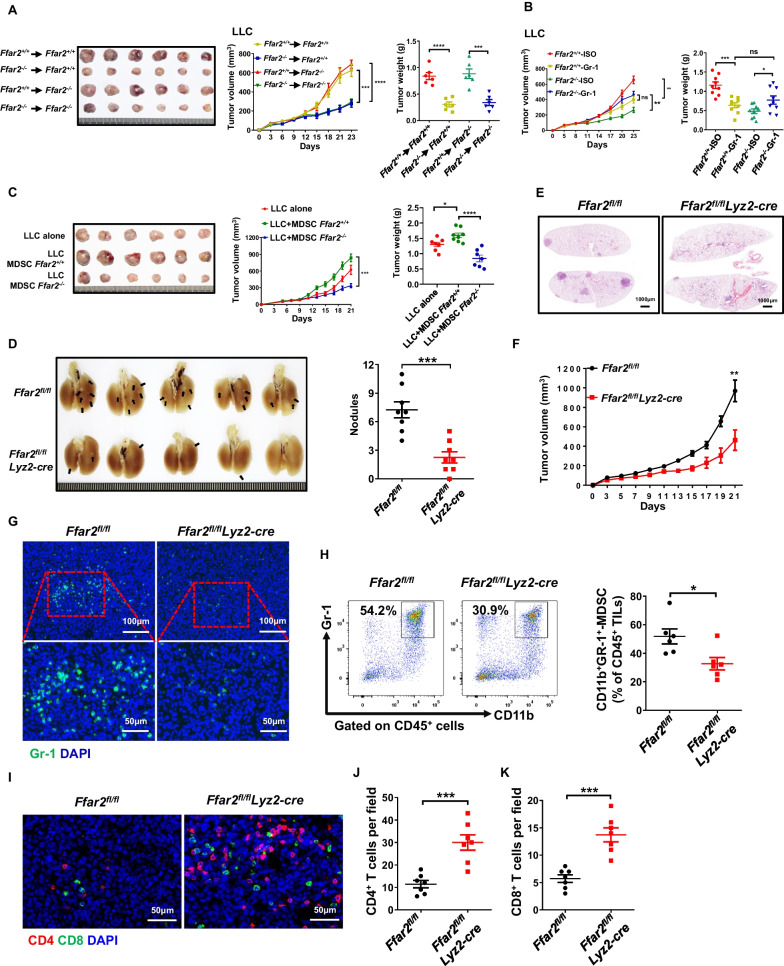
FFAR2 expressing MDSCs contribute to cancer progression. **A** Lethally irradiated *Ffar2*^+*/*+^ mice received BMs transplants from *Ffar2*^+*/*+^ or *Ffar2*^*−/−*^ mice and lethally irradiated *Ffar2*^*−/−*^ mice received BMs transplants from *Ffar2*^+*/*+^ or *Ffar2*^*−/−*^ mice. Ten weeks after transplantation, chimeric mice were injected subcutaneously with LLC (1 × 10^6^ cells/mice, n = 6, biological replicates). Tumors were photographed. Tumor growth and tumor weight were recorded. **B** Mice bearing LLC tumors (n = 6, biological replicates) were injected intraperitoneally with isotype or anti-Gr-1 antibodies (200 μg/mouse, every 4 days). Tumor growth and tumor weight were recorded. **C** Tumor growth and tumor weight in WT mice injected with LLC cells (5 × 10^5^ cells/mouse, n = 6, biological replicates) or co-injected with LLC cells and *Ffar2*^+*/*+^ MDSCs (5 × 10^5^:5 × 10^5^, n = 6, biological replicates) or co-injected with LLC cells and *Ffar2*^*−/−*^ MDSCs (5 × 10^5^:5 × 10^5^, n = 6, biological replicates). MDSCs were isolated from the spleen of LLC tumor-bearing mice and tumor growth was recorded. **D** Mice received weekly intraperitoneal (*i.p.*) injections of urethane (1 g/kg body weight) for 10 weeks, and lung tumor nodules in mice were photographed and quantified (n = 8, biological replicates). **E** Representative histopathology of tumor-bearing lungs was analyzed by H&E staining (n = 5, biological replicates). **F** LLC tumor growth was recorded (n = 6, biological replicates). **G** Immunofluorescence analysis of infiltrating Gr-1^+^-MDSC in LLC tumors. **H** The percentage of tumor-infiltrating CD11b^+^Gr-1^+^-MDSC of total CD45^+^ tumor-infiltrating leukocytes (TILs) in tumors of *Ffar2*^*fl/fl*^ and *Ffar2*^*fl/fl*^*Lyz2-cre* mice were analyzed by flow cytometry (n = 6, biological replicates). I-K, Representative images of multicolored immunofluorescence staining for CD4^+^ and CD8^+^ in LLC tumors (**I**). CD4^+^ T cells (**J**) (n = 7, biological replicates), and CD8^+^ T cells (**K**) (n = 7, biological replicates) were quantified. All data are shown as mean ± SEM, and the experiment was performed three times and a representative example is shown. **A**–**C** were analyzed by two-way ANOVA. **D**, **F**, **H**, **J** and **K** were analyzed by unpaired Student's *t*-test (*P < 0.05, **P < 0.01, ***P < 0.001, ****P < 0.0001 and NS, not significant)

Especially, the growth of LLC tumors was markedly restrained in mice reconstituted with *Ffar2*^−/−^ bone marrow cells as compared to that reconstituted with *Ffar2*^+/+^ bone marrow cells (Fig. 4A). FFAR2 deficiency restricted tumor growth was eliminated by depletion of MDSCs (Fig. 4B) and the infiltration of MDSCs, CD4, CD8, DCs, NKs and macrophages in LLC tumor-bearing mouse upon isotype and anti-Gr-1 antibody treatment was shown in Additional file 1: Fig. S5A–F, implying the dominant role of FFAR2 in the protumoral phenotypes of MDSCs. Moreover, the tumor growth was little changed between co-injection of LLC with wild-type and* Ffar2*^*−/−*^ macrophages (Additional file 1: Fig. S6A). Additionally, co-injection of *Ffar2*^*−/−*^ MDSCs markedly delayed tumor growth than *Ffar2*^+*/*+^ MDSCs both in LLC (Fig. 4C) and B16F10 (Additional file 1: Fig. S6B and C) tumor models. Furthermore, the number and size of urethane-induced nodules were dramatically decreased in the *Ffar2*^*fl/fl*^*Lyz2-cre* mice which FFAR2 was specifically depleted in myeloid cells (Fig. 4D, E). Moreover, the subcutaneous LLC and B16F10 tumor growth was also restrained in *Ffar2*^*fl/fl*^*Lyz2-cre* mice (Fig. 4F and Additional file 1: Fig. S6D). Immunofluorescent staining and flow cytometry demonstrated that conditional knockout of *Ffar2* in myeloid cells led to much lower MDSCs infiltration (Fig. 4G, H), but significantly enhanced CD4^+^ and CD8^+^ T cell infiltration (Fig. 4I–K and Additional file 1: Fig. S6E). These data suggested the great potential of FFAR2 as a specific target for MDSCs in reshaping immune suppressive TME.

### FFAR2 deletion attenuates the immunosuppressive activity of MDSCs

**Fig. 5:**
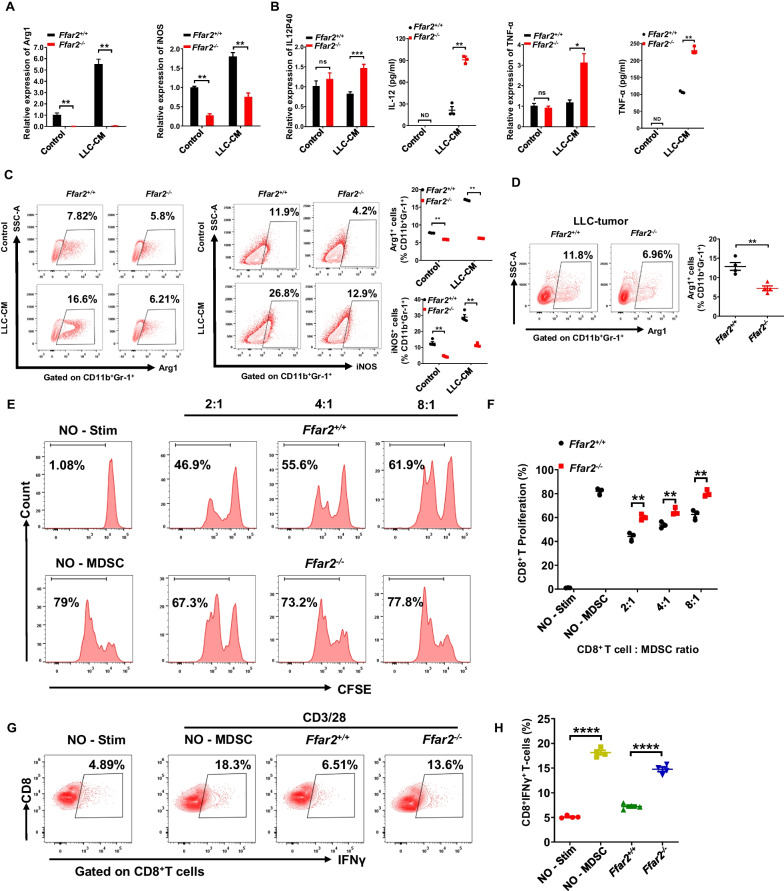
FFAR2 deletion decreases the immune suppressive activity of MDSCs. **A**–**C** *Ffar2*^+*/*+^ and *Ffar2*^*−/−*^ bone marrow-derived MDSCs (1 × 10^6^ cells/well) were seeded in a 6-well plate and cultured in complete RPMI 1640 medium overnight. After overnight resting, BM-MDSCs were re-stimulated by combination GM-CSF (40 ng/ml) with IL-6 (40 ng/ml) or by in the presence of LLC-TES (30%) for 24 h. **A** Relative mRNA levels of Arg1 and iNOS in bone marrow-derived *Ffar2*^*−/−*^ MDSCs compared to *Ffar2*^+*/*+^ MDSCs, determined by real-time RT-PCR (n = 3, biological replicates). **B** Relative mRNA and protein levels of IL12 and TNF-α in bone marrow-derived *Ffar2*^*−/−*^ MDSCs compared to *Ffar2*^+*/*+^ MDSCs, determined by real-time RT-qPCR or ELISA (n = 3, biological replicates). **C** Representative gating strategy and the percentage of Arg1^+^ and iNOS^+^ cells in bone marrow-derived MDSCs (n = 3) were analyzed by flow cytometry (n = 3, biological replicates). **D** Representative gating strategy and the percentage of Arg1^+^ cells in LLC tumor-infiltrating MDSC was analyzed by flow cytometry (n = 4, biological replicates). **E** and **F** Suppression of T cell proliferation in MDSCs isolated from *Ffar2*^+*/*+^ or *Ffar2*^*−/−*^ tumor-bearing mice spleen. MDSC-CD8^+^ T cell (CFSE labelled T cells) suppression assay was analyzed by flow cytometry (**E**), and quantified (**F**) (n = 3, biological replicates). **G** and **H**, IFNγ expression of CD8^+^ T cell in co-culture with MDSCs from *Ffar2*^+*/*+^ or *Ffar2*^*−/−*^ tumor-bearing mice spleen was shown by flow cytometry (**G**), and the percentage of CD8^+^IFNγ^+^ T-cells cells in total CD8^+^ T cells was quantified (**H**) (n = 4, biological replicates). **A**–**D**, **F** and **H** are shown as mean ± SEM, and the experiment was performed three times and a representative example is shown. **A**–**D**, **F** and **H** were analyzed by unpaired Student's *t*-test (*P < 0.05, **P < 0.01, ***P < 0.001, ****P < 0.0001 and NS, not significant)

Then, bone marrow derived MDSCs (BM-MDSCs) were generated [4, 22] and activated using GM-CSF plus IL-6 with or without LLC tumor explant supernatants. We found FFAR2 deficiency did not affect the maturation, proliferation and apoptosis of BM-MDSCs (Additional file 1: Fig. S6F–H). However, key suppressive effectors including Arg1 and iNOS [5] were significantly reduced while pro-inflammatory factors including IL12-p40 and TNF-α were markedly elevated in *Ffar2*^*−/−*^ BM-MDSCs (Fig. 5A–C). Similar outcomes as well as reduced IL-10 expression were also observed in splenetic MDSCs of LLC tumor-bearing mice (Additional file 1: Fig. S6l–K). Furthermore, flow cytometry showed that percentage of Arg1^+^ cells were markedly reduced in LLC tumor derived-MDSCs (Fig. 5D) and urethane-induced lung cancer tissues of *Ffar2*^*−/−*^ mice (Additional file 1: Fig. S7A). Meanwhile, the proliferation (Fig. 5E, F, and Additional file 1: Fig. S7B and C) and IFNγ production (Fig. 5G, H, Additional file 1: Fig. S7D and E) was also enhanced significantly in *Ffar2*^*−/−*^ MDSCs treated T cells. As a consequence, the producing of IFNγ and TNF-α by CD8^+^ T cells from tumors was notably increased in *Ffar2*^*fl/fl*^*Lyz2-cre* mice (Additional file 1: Fig. S7F). Collectively, these data suggest that FFAR2 deletion impairs the immunosuppressive activity of MDSCs on T cells in the tumor microenvironment.

### FFAR2 reprograms MDSCs through Gαq/Calcium/PPAR-γ signaling pathway

**Fig. 6:**
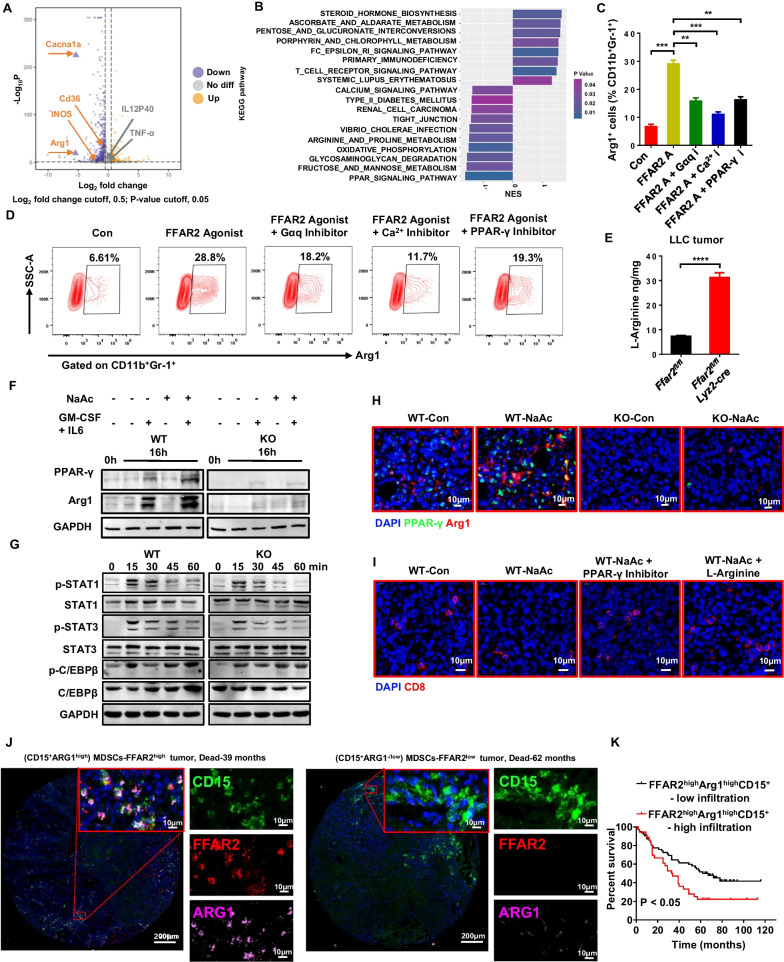
FFAR2 upregulates Arg1 in MDSCs through G_αq_/Calcium/PPAR-γ signaling pathway. **A** Volcano plot showed significant differences in gene expression of *Ffar2*^*−/−*^ MDSC compared with *Ffar2*^+*/*+^ MDSC cells (n = 3, biological replicates). (Downregulated genes, purple; Upregulated genes, orange). Interested differentially expressed genes were shown in triangle mark (downregulated) and square mark (upregulated). **B** All significant KEGG (Kyoto Encyclopedia of Genes and Genomes) enrichment analysis results from the *Ffar2*^*−/−*^ MDSC compared with *Ffar2*^+/+^ MDSC (*P* ≤ 0.05). NES: normalized enrichment score. **C** and **D** BM-MDSCs were pretreated with DMSO (0.1%), FFAR2 Agonist (10 μM), G_αq_ inhibitor (YM-254890; 1 μM), Ca^2+^ inhibitor (2-APB; 100 μM) and PPAR-γ-inhibitor (GW9662; 2 μM) for 2 h before adding GM-CSF and IL-6. All MDSCs were treated for 24 h, and the percentage of Arg1^+^ cells (**C**) and representative gating strategy (**D**) were analyzed by flow cytometry (n = 3, biological replicates). **E** Quantification of L-Arginine in LLC tumor tissue extracts. **F** BM-MDSCs were activated by GM-CSF and IL6 with or without NaAc. The expression of PPAR-γ, Arg1 and GAPDH were detected by Western blotting. **G** BM-MDSCs were activated by GM-CSF and IL6 for the indicated time. The expression of GAPDH, p-STAT1 (Tyr701), STAT1, p-STAT3 (Tyr705), STAT3, p-C/EBPβ (Thr217) and C/EBPβ were detected (n = 3, biological replicates). **H** Mice bearing LLC tumors (n = 3, biological replicates) and received intraperitoneal (*i.p.*) injections of PBS or NaAc. Representative images of immunofluorescence staining for PPAR-γ and Arg1 in tumors. **I** LLC-tumor bearing mice were treated with NaAc (500 mg/kg), NaAc + PPAR-γ inhibitor (1 mg/kg), NaAc + L-Arginine (1.5 mg/kg). Representative images of immunofluorescence staining for CD8 in tumors. **J** and **K** Representative images of multicolor immunofluorescence staining of a lung adenocarcinoma patient with high FFAR2 expression in (CD15^+^ARG1^high^)-MDSCs (Left, who was still alive 39 months after surgery) and low FFAR2 expression in (CD15^+^ARG1^−/low^)-MDSCs (Right, who was still alive 62 months after surgery) (**J**). Log-rank (Mantel-Cox) test of cumulative survival rates of lung adenocarcinoma patients subdivided by the percentage FFAR2^high^Arg1^high^CD15^+^-MDSC of total cells (with > 3%, high infiltration; with ≤ 3%, low infiltration) in tumor tissues (**K**). **C** and **E** are shown as mean ± SEM, and the experiment was performed three times and a representative example is shown. **C** and **E** were determined by unpaired Student's *t-*test (*P < 0.05, **P < 0.01, ***P < 0.001, ****P < 0.0001 and NS, not significant)

After analyzing the transcriptome profile of BM-MDSCs by RNA sequencing (RNA-seq), 737 down-regulated 301 up-regulated genes were identified in *Ffar2*^*−/−*^ BM-MDSCs. In particular, key immunosuppressive genes Arg1 and NOS2 were significantly down-regulated in *Ffar2*^*−/−*^ BM-MDSCs (Fig. 6A). In addition, KEGG pathway gene set enrichment analysis revealed signaling pathways associated with calcium, PPAR and arginine metabolism were significantly changed in *Ffar2*^*−/−*^ BM-MDSCs, which may result in enhanced L-Arginine and T cell anti-tumor responses [23] (Fig. 6B). In order to explore the correlation between FFAR2, PPAR-γ and Arg1, we treated the MDSCs with indicated agonists or inhibitors. As shown in Fig. 6C, D, and Additional file 1: Fig. S8A, both the FFAR2 ligand (NaAc) and agonist upregulated Arg1 expression, while PPAR-γ-inhibitor (GW9662), Gαq-inhibitor (YM-254890) and Ca^2+^-inhibitor (2-APB) markedly suppressed the increased Arg1 expression, indicating that FFAR2 promoted Arg1 expression depended on Gαq/Calcium/PPAR-γ signaling pathway. Accordingly, the accumulation of L-Arginine in the tumor from *Ffar2*^*fl/fl*^*Lyz2-cre* mice was enhanced significantly (Fig. 6E), which is consistent with the reduced consumption of L-Arginine in *Ffar2*^*−/−*^ BM-MDSCs (Additional file 1: Fig. S8B). Furthermore, only the expression of PPAR-γ and Arg1 but not other key transcription factors such as STAT1, STAT3 and C/EBPβ was dramatically reduced in *Ffar2*^*−/−*^ MDSCs (Fig. 6F and G, and Additional file 1: Fig. S8C and D) and urethane-induced lung cancer tissues (Additional file 1: Fig. S8E). The expression of PPAR-γ and Arg1 are increased significantly by FFAR2 ligand (NaAc) in the LLC tumors from *Ffar2*^+*/*+^ mice. Whereas, the tumors from *Ffar2*^*−/−*^ mice were little influenced (Fig. 6H). Accordingly, the NaAc reduced CD8^+^ T cell infiltration was eliminated by PPAR-γ-inhibitor (GW9662) and L-Arginine, suggesting the predominant role of L-Arginine in T cell mediated antitumor immunity (Fig. 6I). In addition, much more FFAR2^high^ARG1^high^CD15^+^-MDSCs were observed in the tumor of lung cancer patients with short survival compared to that with longer survival (Fig. 6J, K). Collectively, these data suggest the predominant role of Gαq/Calcium/PPAR-γ/Arg1 signaling as well as L-Arginine consumption in FFAR2 mediated immune suppression, which have great potential to be targets for cancer immunotherapy.

### Therapeutic targeting of FFAR2 enhanced anti-PD1 therapy

**Fig. 7:**
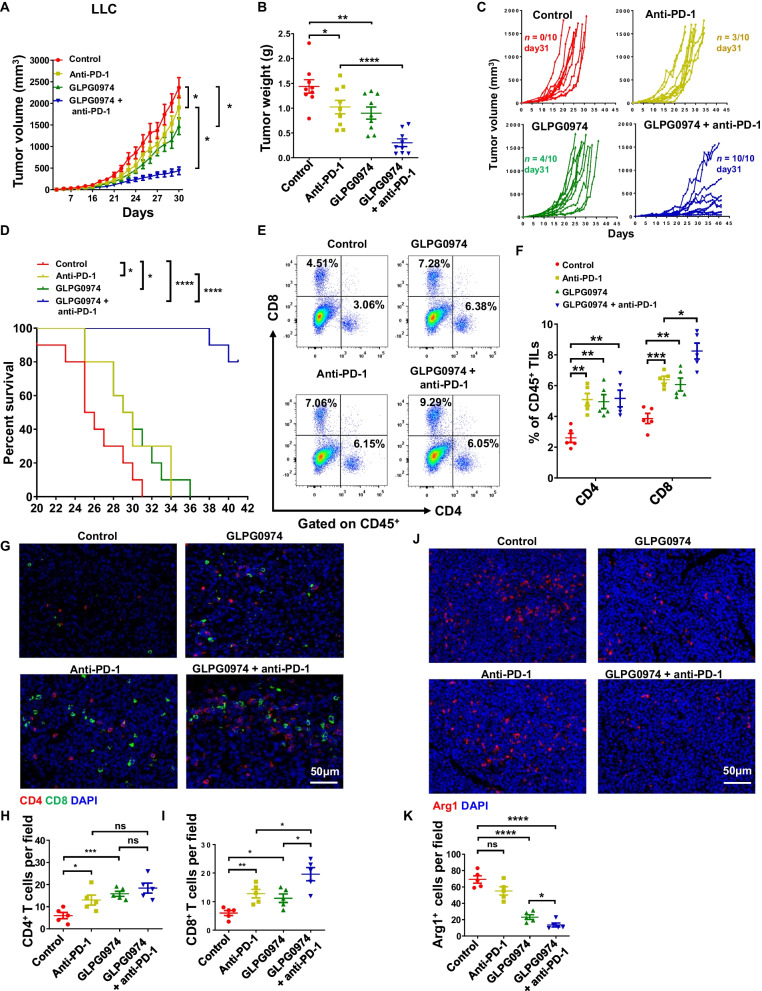
FFAR2 inhibitor enhances the effect of anti-PD1 therapy. **A**–**E** WT mice were injected subcutaneously with LLC cells (1 × 10^6^ cells/mouse) and treated with FFAR2 inhibitor (GLPG0974, 5 mg kg^−1^ per day; single esophageal gavage), intraperitoneally anti-PD1 (200 μg/mouse; every 4 days), FFAR2 inhibitor (GLPG0974) + anti-PD1 and PBS as control. Therapy was started at day 4 after tumor inoculation. LLC tumor growth (A) (n = 9) and tumor weight (**B**) (n = 9) were recorded. **C** and **D** Individual tumor growth of subcutaneous LLC tumors were recorded, and the number of survival tumor-bearing mouse at day 31 was shown (**C**) (n = 10), and overall survival of LLC tumor-bearing mice was recorded (Log-rank (Mantel-Cox) test) (**D**) (n = 10). **E** and **F** Representative gating strategy (**E**) and the percentage (**F**) of tumor-infiltrating CD4^+^ or CD8^+^-T cell of total CD45^+^ tumor-infiltrating leukocytes (TILs) in LLC tumors on day 30 post implantation were analyzed by flow cytometry (n = 5). **G**–**I**, Representative images of multicolor immunofluorescence staining for CD4 and CD8 in mouse LLC tumors (**G**). CD4^+^ T cells (**H**) (n = 5), and CD8 T cells (**I**) (n = 5) were quantified. J and K, Representative images of immunofluorescence staining for Arg1 in mouse LLC tumors (**J**). Arg1^+^ cells were quantified (**K**) (n = 5). **A**, **B**, **F**, **H**, **I** and **K** are shown as mean ± SEM, and the experiment was performed three times and a representative example is shown. **A**, **B**, **F**, **H**, **I** and **K** were analyzed by one-way ANOVA. (*P < 0.05, **P < 0.01, ***P < 0.001, ****P < 0.0001 and NS, not significant)

To evaluate the potential of FFAR2 inhibition in immune checkpoint therapy, we treated tumor bearing mice with FFAR2 inhibitor (GLPG0974) with or without anti-PD-1 antibody. Although the LLC tumor growth was restrained by FFAR2 inhibitor and anti-PD-1 antibody respectively, the combination therapy dramatically enhanced this inhibition on both tumor size and weight (Fig. 7A–C), and extended the survival of tumor-bearing mice (Fig. 7D). Then, we dissected LLC tumors to analyze tumor-infiltrating leukocytes (TILs) at day 30. Flow cytometry and immunofluorescent staining showed that the infiltrating CD8^+^ T cells was significantly increased in tumors from combination therapy group mice (Fig. 7E–I). Which is consistent with in vitro data, both FFAR2 inhibitor and combination therapy (FFAR2 inhibitor plus anti-PD-1 antibody) downregulated the expression of Arg1 in LLC tumors (Fig. 7J, K). Moreover, immunofluorescent staining showed that the infiltrating Gr-1^+^-MDSC was significantly decreased in tumor from FFAR2 inhibitor or combination therapy group (Additional file 1: Fig. S9A–C). Above data suggest that FFAR2 deletion or pharmaceutical inhibition could be a new strategy to overcome resistance to PD-1/PD-L1 blockade in lung cancer immunotherapy.

## Discussion

As the abundant leukocytes in human blood, MDSCs are essential for restricting adaptive immune cells, in particular, cytotoxic T lymphocytes in tumor immune evasion. Whereas, the biological characteristics and immunosuppressive mechanisms of MDSCs in cancer development and immune evasion remained elusive. Here, we demonstrated an extraordinary and particular regulation to MDSCs by aberrant cancer metabolism pathway. The accumulation of tumor-cell-derived acetic acids exacerbates the immune suppression of FFAR2-expressing MDSCs through up-regulation of Arg1 expression and L-Arginine consumption in tumor microenvironment, which contribute to immune suppression and cancer progression in a Gαq/Calcium/ PPAR-γ/Arg1 signaling dependent manner. Furthermore, FFAR2 deletion, pharmaceutical inhibition or L-Arginine supplementation reverse the immunosuppressive microenvironment and promote T cell infiltration and anti-tumor response significantly, which have the great potential to be a novel target to enhance the outcomes of cancer immunotherapy.

The elevated level of glycolysis of tumors provides both energy and precursors for fatty acid synthesis, and accompanied with a coordinated rise in lipogenic and glycolytic enzyme activities [24]. Most tumors and their precursor lesions unexpectedly undergo exacerbated endogenous fatty acid biosynthesis irrespective of the levels of extracellular lipids [25]. Accordingly, ACLY (ATP citrate lyase) and FASN (fatty acid synthetase) which are involved in de novo fatty acid synthesis are both greatly upregulated in cancer cells. Moreover, FASN overexpression and hyperactivity commonly occurs in carcinomas with higher risk of both disease recurrence and death [25, 26]. Whereas, ACLY inhibition reduces tumorigenesis in vivo, implying the importance of fatty acid biosynthesis in tumor formation. Here, we demonstrated a significant accumulation of short chain fatty acids (especially acetic acids) in both human and mouse tumor tissues, as well as tumor cells’ supernatant. Previous studies of SCFAs/FFAR2 signaling are most focused on gut microbiota mediated immune regulation colorectal cancer model. However, the role of FFAR2 in tumor immune evasion through cancer cells exacerbated endogenous fatty acids remains unclear. Here we showed the fundamental role of extraordinary accumulated acetic acids in cancer cells induced immune evasion through consuming L-Arginine in tumor microenvironment by FFAR2^+^ MDSCs. Whole or myeloid *Ffar2* gene deletion markedly inhibited urethane-induced lung carcinogenesis, Lewis lung carcinoma (LLC), and B16F10 melanoma tumor growth, through restricting MDSCs mediated L-Arginine consumption and promoting CD8^+^ T cell infiltration as well as anti-tumor response in the tumor microenvironment which benefit the outcome of cancer immunotherapy significantly. Taken together, our study explored a novel linker between extraordinary fatty acids biosynthesis of cancer cells and L-Arginine consumption by MDSCs in immune evasion and identified a new target to metabolic reprogram for cancer immunotherapy.

MDSCs are present at all stages of tumor growth, which strongly inhibit CD8^+^ T cell infiltration and antitumor responses in TME [27–29]. Although PMN-MDSCs, pathologically activated neutrophils, represent the most abundant population of MDSCs, it is still hard to define these cell population through current surface markers due to heterogeneity of MDSCs [30, 31]. As the most important metabolite-sensing receptor, FFAR2 also known as GPR43, exerts immunomodulatory effects and functions in gut homeostasis and the regulation of inflammation by altering leukocyte chemotaxis and colonic regulatory T (Treg) cell expansion [32, 33]. However, the role of FFAR2 in tumor infiltrated immune cells remains unknown. To explore the target immune cells involved in acetic acids mediated tumor immune evasion, clinical tumor tissue array and tumor-bearing mouse spleen were analyzed, and found that FFAR2 highly expressed in human tumor derived and tumor-bearing mouse splenic MDSCs. Moreover, tumor-infiltrating immune cells analysis showed that whole or myeloid *Ffar2* gene deletion markedly reduce MDSCs accumulation, but increases CD4^+^ and CD8^+^ T cell infiltration in tumors. Furthermore, MDSCs deletion in vivo, co-injection of MDSCs with tumor cells and myeloid cell conditional knockout *Ffar2* mouse tumor models showed that FFAR2 drive tumor progression mainly through MDSCs. Thus, FFAR2 was demonstrated to be a key regulator for pro-tumor MDSCs in reshaping immune suppressive TME.

MDSCs mediated immune suppression are also driven by signal transducer and activator of transcription (STAT1/3) and CCAAT/enhancer-binding protein-β (C/EBPβ) regulated Arg1 and inducible nitric oxide synthase (iNOS) [34, 35]. The gene set enrichment analysis (GSEA) of RNA-seq revealed that FFAR2 deficiency markedly downregulated the calcium and PPAR-γ signaling pathway, as well as the expression of Arg1. Although previous study demonstrated that microbiota-derived butyrate breaks balance of Treg/Th17 cells through PPAR-γ signaling, the FFARs involved in Arg1 and arginine metabolism was not shown [36]. Most importantly, L-Arginine concentrations directly impact the metabolic fitness and survival capacity of T cells that are crucial for anti-tumor responses [23]. And metabolic modulation of L-Arginine has been proven to have great clinical potential in enhancing the efficacy of immunotherapies [37].

Here, we demonstrated that PPAR signal pathway mediated Arg1 expression were almost completely eliminated in FFAR2^−/−^ MDSCs. Furthermore, the NaAc or FFAR2 agonist-induced PPAR-γ signaling and Arg1 expression was blocked significantly by PPAR-γ antagonist (GW9662) as well, suggesting the dominated role of PPAR-γ in MDSCs mediated T cell suppression by Arg1. Furthermore, not only the accumulation of L-Arginine was enhanced significantly in the tumor from FFAR2 conditional knock mice, but also the consuming of L-Arginine was hampered significantly in FFAR2 knockout MDSCs. And replenishment of L-Arginine or inhibition to PPAR-γ increase the infiltration and antitumor immunity of T cell significantly. Although COX2 inhibitors could reduce the expansion and block the Arg1 expression of MDSCs as well [31, 38, 39], long-term systemic use of COX2 inhibitors endowed with severe side effects [31]. Most importantly, these findings suggested that the FFAR2 expressing MDSCs subset play crucial roles in mutual regulation of fatty acid and arginine metabolism. Moreover, the Acetic acids/FFAR2 axis enhanced the expression of Arg1 through the Calcium/PPAR-γ pathway, suggesting inhibitors or modulators targeting Calcium/PPAR-γ or SCFA excess in combination with anti-PD-1 antibodies will have potential clinical value in improving cancer treatment. In summary, our study suggested a novel strategy to eliminate the pathologically activated MDSCs specifically by targeting FFAR2 without excessive off-target effects which have great potential in clinical application for cancer immunotherapy.

## Conclusions

In this study, we disclose the accumulation of acetic acids in TME, the tumor cell metabolites to enhance the immunosuppressive activity of FFAR2 expression MDSCs. Whole or myeloid *Ffar2* gene deletion markedly restrained tumor progression, and impairs the immunosuppressive activity of MDSCs to reconstitute a more anti-tumoral TME. The combination therapy strategy of FFAR2 pharmaceutical inhibition with anti-PD-1 antibody demonstrated superior antitumor effectiveness to anti-PD-1 antibody therapy alone in LLC subcutaneous mouse tumor model. These findings imply that FFAR2 may serve as a potential new target to eliminate pathologically activated MDSCs, and contribute to clinical outcomes of cancer immunotherapy.

### Supplementary Information

**Additional file 1.** Supplementary figures S1–S9.

**Additional file 2.** Primers used for qPCR.

## Data Availability

All data needed to evaluate the conclusions in the paper are present in the manuscript or the Additional file 1.

## Abbreviations

Arg1: Arginase-1
ATCC: American type culture collection
BM-MDSCs: Bone marrow derived MDSCs
C/EBPβ: CCAAT/enhancer-binding protein beta
CFSE: Carboxyfluorescein succinimidyl ester
CTL: Cytotoxic T lymphocytes
FFAR2: Free fatty acid receptor 2
FFAs: Free fatty acids
FAO: Fatty acid oxidation
FFARs: Free fatty acid receptors
GC–MS: Gas chromatography–mass spectrometry
GPCRs: G protein-coupled receptors
GM-CSF: Granulocyte-macrophage colony-stimulating factor
H&E: Hematoxylin and eosin
HUVEC: Human umbilical vein endothelial cell
HSCs: Hematopoietic stem cells
IL6: Interleukin 6
ICB: Immune checkpoint blockade
LUAD: Lung adenocarcinoma
LLC: Lewis lung carcinoma cell
M-MDSCs: Monocytic MDSCs
PPAR-γ: Peroxisome proliferator-activated receptor gamma
PMN-MDSCs: Granulocytic/polymorphonuclear MDSCs
SCFAs: Short-chain fatty acids
STAT1/3: Signal transducer and activator of transcription 1/3
TME: Tumor microenvironment
