# Supplementary material for: FFAR2 expressing myeloid-derived suppressor cells drive cancer immunoevasion
Source: J Hematol Oncol. 2024 Feb 24;17:9. doi: 10.1186/s13045-024-01529-6 (PMC10894476; doi:10.1186/s13045-024-01529-6)
Supplement: Supplementary file 1 — Additional file 1. Supplementary figures S1–S9. [file 13045_2024_1529_MOESM1_ESM.docx]

Supplementary Data File1 for:

**FFAR2 expressing myeloid-derived suppressor cells drive cancer immunoevasion**

**Authors:** Zeda Zhao^1#^, Juliang Qin^1#^, Ying Qian^1^, Chenshen Huang^2^, Xiaohong Liu^1^, Ning Wang^3^, Liqin Li^3^, Yuqing Chao^1^, Binghe Tan^4^, Na Zhang^4^, Min Qian^1^, Dali Li^1^, Mingyao Liu^1^, Bing Du^1^*

**Supplementary Figures S1-S9 and Figure Legends**


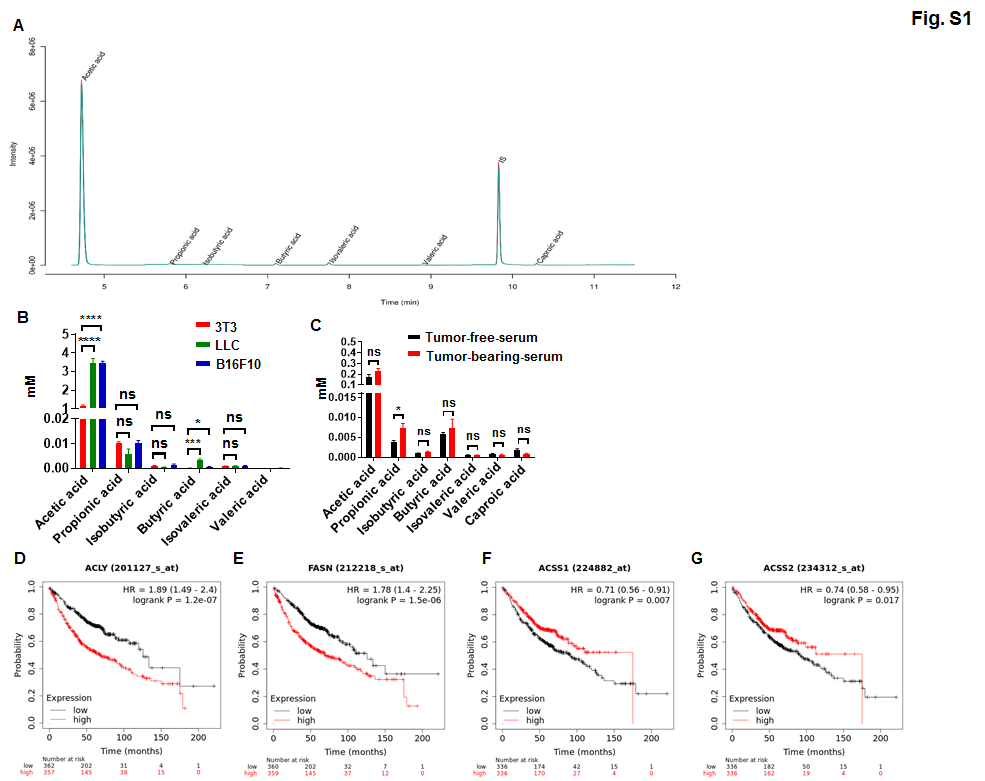


**Supplementary Figure 1. SCFAs’ concentration in mouse serum and mouse cell cultures, and the correlation of fatty acid metabolism-related genes expression with lung adenocarcinoma patient’s prognostic.**

(A) Detecting peaks of gas chromatography-mass spectrometry (GC-MS) for supernatants from 16 h cultures of human tumor cells. (B) Quantification of SCFAs in supernatants from 16h cultures of mouse normal or tumor cells (1 × 10^6^ cells/well, n = 3). (C) LLC cells were injected subcutaneously into C57BL/6 mice (1 × 10^6^ cells/mouse, n = 4). After 16 days, the serum of tumor-free or tumor-bearing mice was collected and analyzed by GC-MS. Quantification of SCFAs in tumor-free or tumor-bearing mice serum was shown (n = 4). D-G, The prognostic significance of FASN, ACSS1, ACSS2 and ACLY in lung adenocarcinoma patients was assessed via Kaplan-Meier analysis. B and C, Data are shown as mean ± SEM, and the experiment was performed three times and a representative example is shown. B was analyzed by one-way ANOVA, and C was analyzed by unpaired Student's *t*-test (*P < 0.05, **P < 0.01, ***P < 0.001, ****P < 0.0001 and NS, not significant).


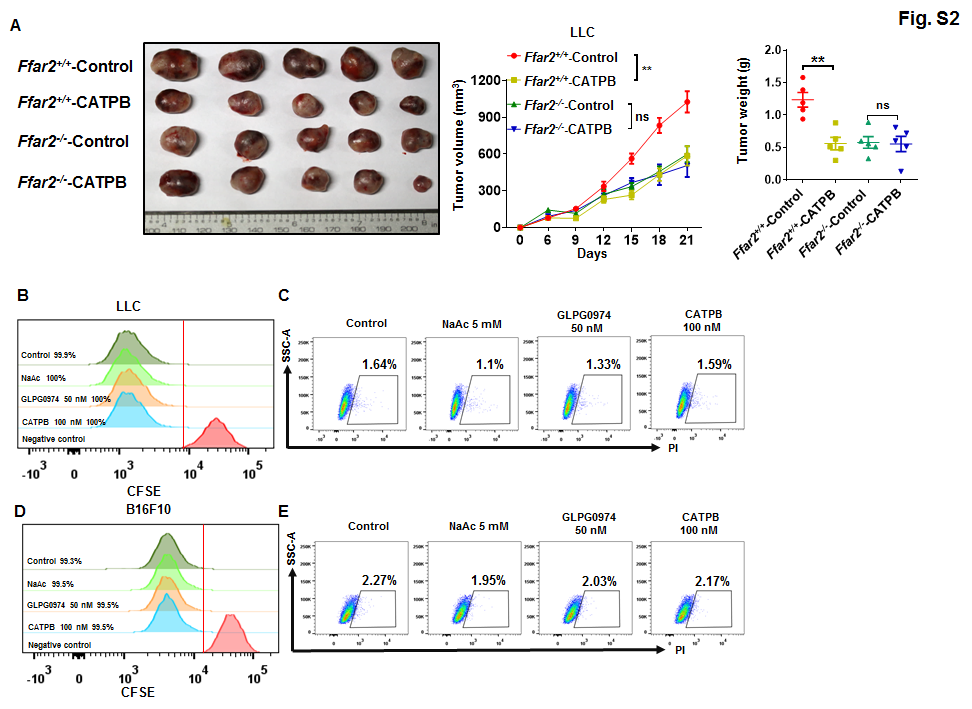


**Supplementary Figure 2. FFAR2 inhibitor delayed the tumor growth in WT mice *in vivo*, but did not influence the proliferation and survival of tumor cells *in vitro*.**

(A) Mice bearing LLC tumors were treated with PBS containing 0.5% DMSO or FFAR2 inhibitor (5mg/kg per day). Tumor growth and tumor weight were recorded (n=5, biological replicates). (B-E) Tumor cells (1 × 10^6^ cells/well) were seeded in a 6-well plate, and treated with DMSO (0.1 %), NaAc (5 mM), FFAR2 inhibitor (GLPG0974, 50 nM) and FFAR2 inhibitor (CATPB, 100nM) for 48 h. The proliferation (B) and survival (C) of LLC cells were analyzed by flow cytometry (n=3, biological replicates). And the proliferation (D) and survival (E) of B16F10 cells were analyzed by flow cytometry (n=3, biological replicates). A was analyzed by two-way ANOVA (*P < 0.05, **P < 0.01, ***P < 0.001, ****P < 0.0001 and NS, not significant).


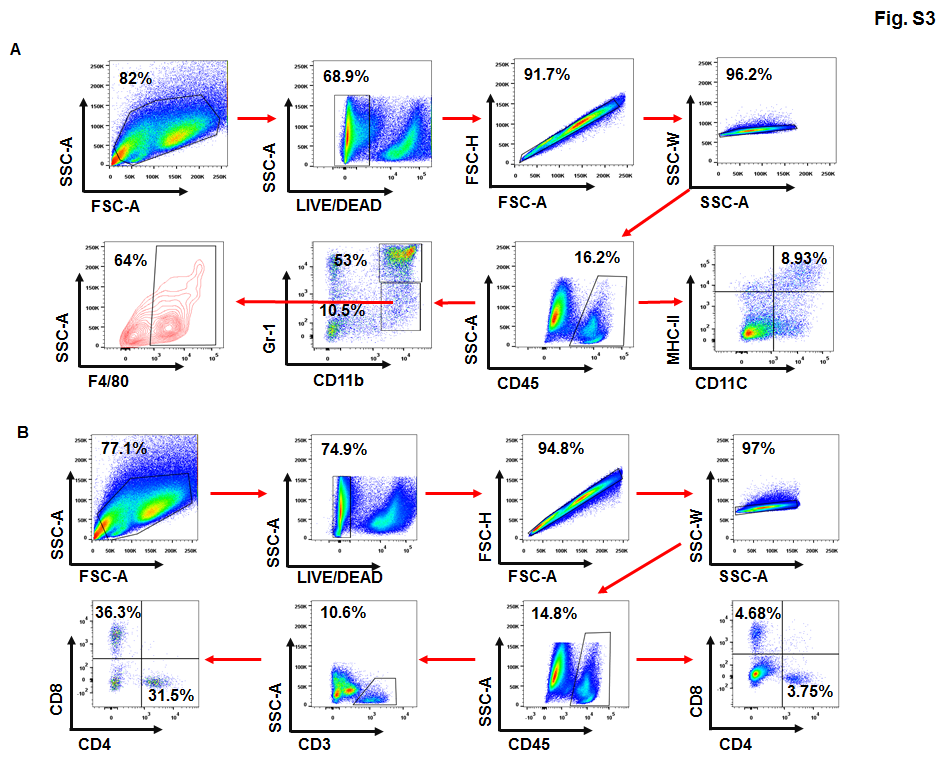


**Supplementary Figure 3. Representative gating strategies of tumor-infiltrating leukocytes (TILs).**

(A) Gating strategy for the quantification of MDSCs, Macrophages and DCs in LLC tumors was analyzed by flow cytometry. (B) Gating strategy for the quantification of T cells in LLC tumors.


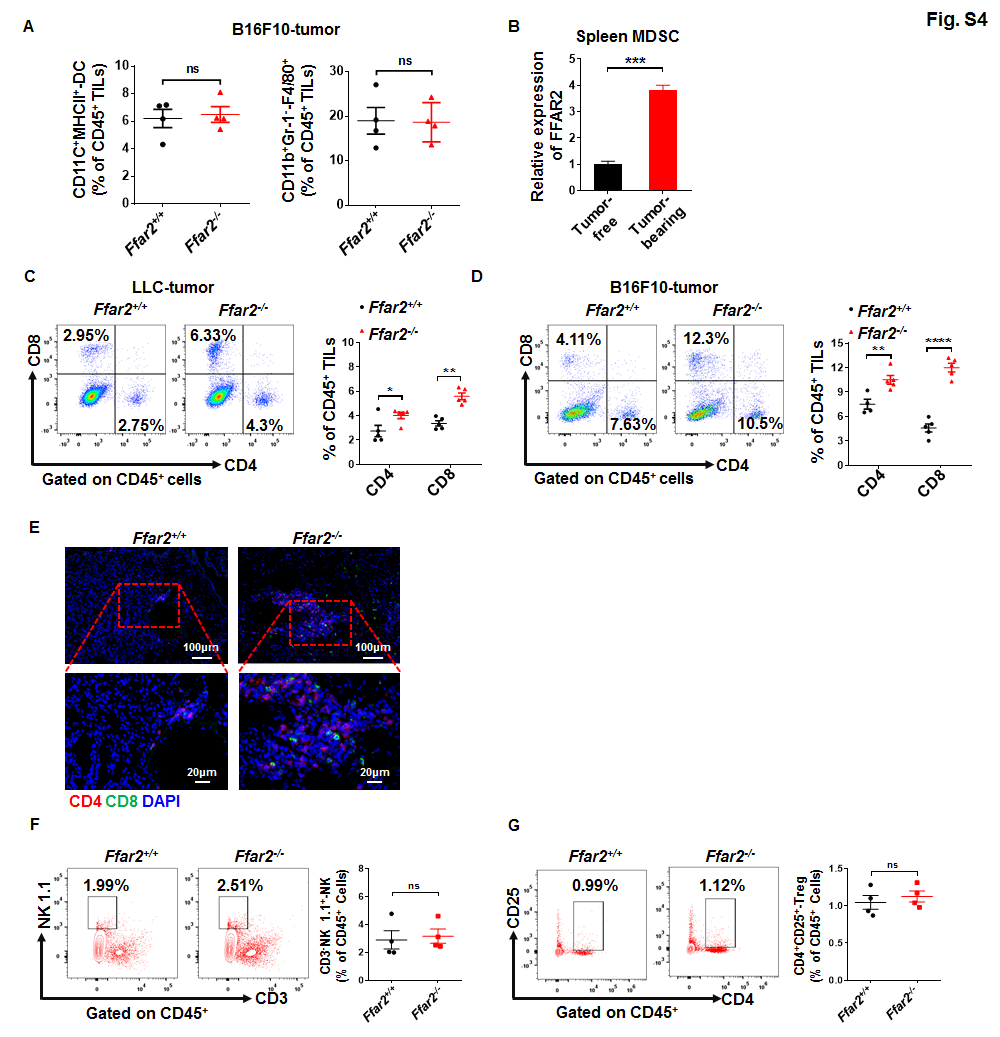


**Supplementary Figure 4. FFAR2 deletion enhances accumulation of T cells in tumor tissues.**

(A) Quantification of percentage of CD11C^+^MHCII^+^-DC and CD11b^+^Gr-1^-^-F4/80^+^-macrophage of total CD45^+^ tumor-infiltrating leukocytes (TILs) in B16F10 tumors (n = 4). (B) LLC cells were injected subcutaneously into C57BL/6 mice. After 16 days, relative FFAR2 expression levels of MDSCs isolated from tumor-free and tumor-bearing mice spleen were determined by real-time RT-qPCR (n = 3). C and D, Representative gating strategy and percentage of tumor-infiltrating CD4^+^ and CD8^+^ T cell of total CD45^+^ tumor-infiltrating leukocytes (TILs) in LLC (C) (n = 5) and B16F10 (D) (n = 5) tumors were analyzed by flow cytometry. (E) Representative images of multicolored immunofluorescence staining for CD4 and CD8 in urethane-induced lung tumor nodules in *Ffar2^+/+^* and *Ffar2^-/-^* mice. F and G, Representative gating strategy and percentage of tumor-infiltrating CD3^-^NK 1.1^+^-NK (F) (n=4, biological replicates) and CD4^+^CD25^+^-Treg (G) (n=4, biological replicates) cell of total CD45^+^ tumor-infiltrating leukocytes (TILs) in LLC. A-D, F and G, Data are shown as mean ± SEM, and the experiment was performed three times and a representative example is shown. A-D, F and G were analyzed by unpaired Student's *t*-test (*P < 0.05, **P < 0.01, ***P < 0.001, ****P < 0.0001 and NS, not significant).


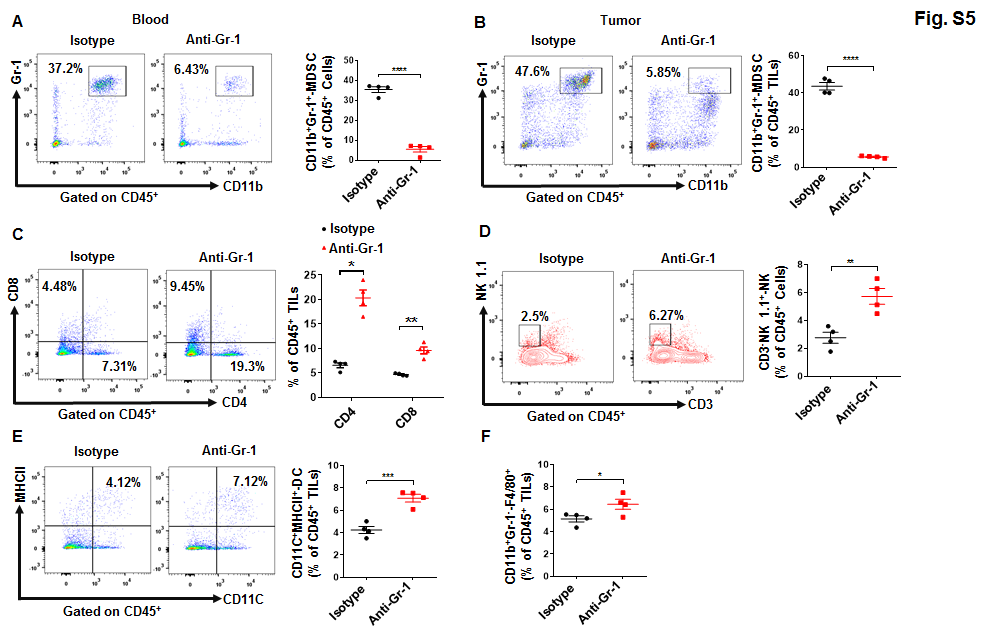


**Supplementary Figure 5. Flow cytometry analyses of leukocytes in LLC tumor-bearing mice.**

(A) Representative gating strategy and percentage of CD11b^+^Gr-1^+^-MDSCs of total CD45^+^ cells in LLC-tumor bearing mouse blood were determined by flow cytometry upon isotype control or anti-Gr-1 treatment (n = 4, biological replicates). B-F, Representative gating strategy and percentage of tumor-infiltrating CD11b^+^Gr-1^+^-MDSCs (B), tumor-infiltrating CD4^+^ and CD8^+^ T cell (C), tumor-infiltrating CD3^-^NK 1.1^+^-NK (D), CD11C^+^MHCII^+^-DC (E) and CD11b^+^Gr-1^-^-F4/80^+^-macrophage (F) upon isotype control or anti-Gr-1 antibody treatment. (n=4, biological replicates). A-F, Data are shown as mean ± SEM, and the experiment was performed three times and a representative example is shown. A-F were analyzed by unpaired Student's *t*-test (*P < 0.05, **P < 0.01, ***P < 0.001, ****P < 0.0001 and NS, not significant).


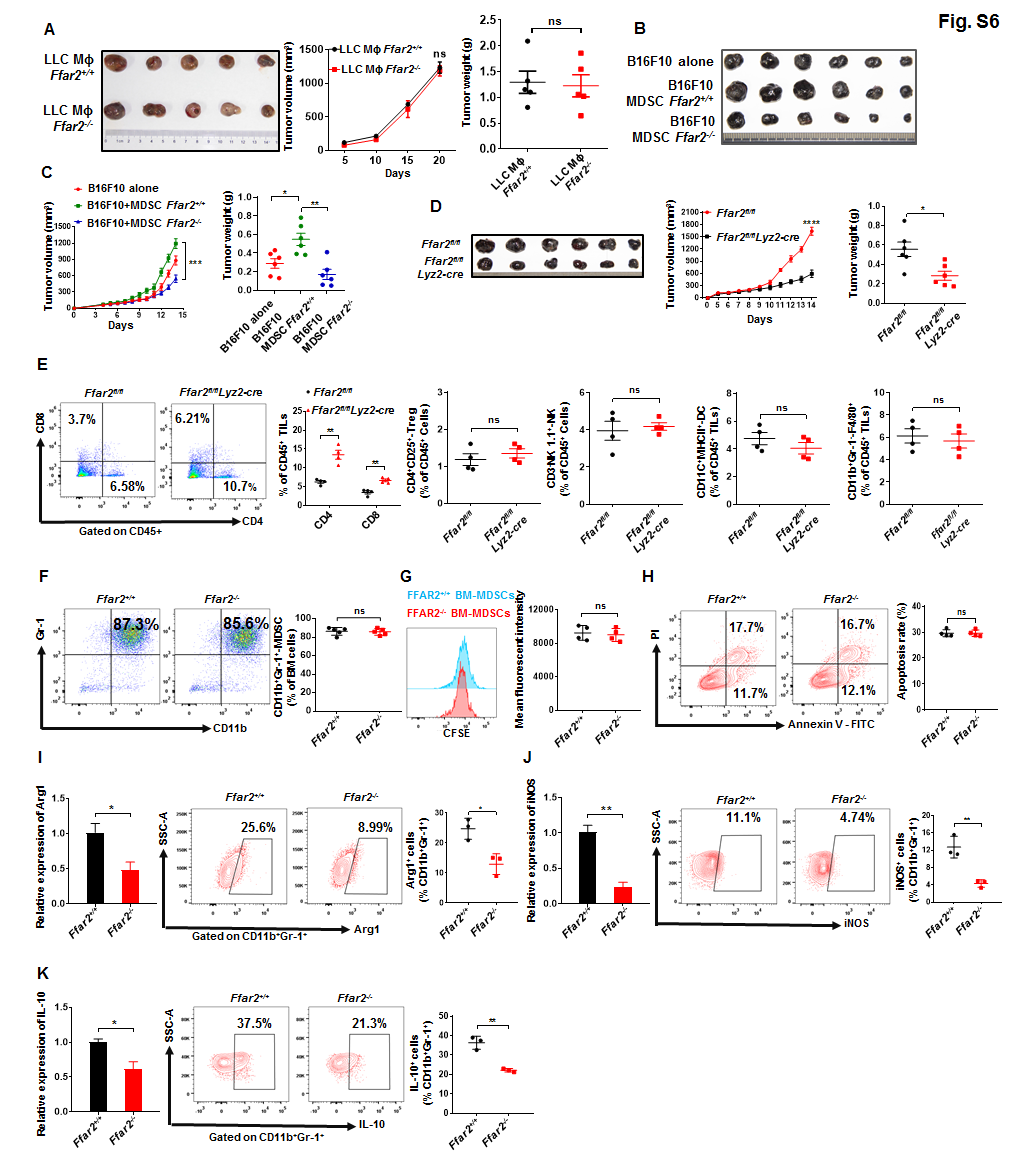


**Supplementary Figure 6. FFAR2 deletion decrease the immune suppressive activity of MDSCs *in vivo*.**

(A) Tumor growth in WT mice co-injected with LLC cells and *Ffar2^+/+^* BMDMs (5 × 10^5^:5 × 10^5^, n = 6-8) or co-injected with LLC cells and *Ffar2^-/-^* BMDMs (5 × 10^5^:5 × 10^5^, n = 6, biological replicates). Primary bone marrow-derived macrophages (BMDMs) from mice were generated as previously described (1). B and C, Tumor growth in WT mice injected with B16F10 cells (5 × 10^5^ cells/mouse, n = 6, biological replicates) or co-injected with B16F10 cells and *Ffar2^+/+^* MDSCs (5 × 10^5^:5 × 10^5^, n = 6, biological replicates) or co-injected with B16F10 cells and *Ffar2^-/-^* MDSCs (5 × 10^5^:5 × 10^5^, n = 6, biological replicates). MDSCs were isolated from the spleen of B16F10 tumor-bearing mice using a MDSC isolation kit. B16F10 tumors were excised and photographed at the end of the experiment (B), and tumor growth and tumor weight were recorded (C). (D) B16F10 cells were injected subcutaneously into *Ffar2^fl/fl^* and *Ffar2^fl/fl^Lyz2-cre* mice (1 × 10^6^ cells/mouse, n = 6, biological replicates). B16F10 tumors were excised and photographed at the end of the experiment. Tumor growth and tumor weight were recorded. (E) The percentage of tumor-infiltrating CD4^+^, CD8^+^, CD4^+^CD25^+^-Treg, CD3^-^NK1.1^+^-NK, CD11C^+^MHCII^+^-DC and CD11b^+^Gr-1^-^-F4/80^+^-macrophage of total CD45^+^ tumor-infiltrating leukocytes (TILs) in tumors of *Ffar2^fl/fl^* and *Ffar2^fl/fl^Lyz2-cre* mice were analyzed by flow cytometry (n = 4, biological replicates). F-H, Bone marrow cells (1 × 10^6^ cells/well) were seeded in a 6-well plate and cultured in presence of GM-CSF (40 ng/ml) and IL-6 (40 ng/ml) for 4 days. After 4 days, the percentage of CD11b^+^Gr-1^+^ cells in BM cells were determined by flow cytometry (F) (n=5, biological replicates). The proliferation (G) and apoptosis (H) of MDSC were analyzed by flow cytometry (n=4, biological replicates). I-K, *Ffar2^+/+^* and *Ffar2^-/-^* mice were injected subcutaneously with LLC cells (1 × 10^6^ cells/mouse). After 21 d of transplantation, single cell suspensions were prepared from tumor-bearing mouse spleen, and MDSCs were isolated from the spleen using a MDSC isolation kit. Relative mRNA and protein levels of Arg1 (I), iNOS (J) and IL-10 (K) were analyzed by real-time RT-qPCR or flow cytometry (n=3, biological replicates). A and C-K are shown as mean ± SEM, and the experiment was performed three times and a representative example is shown. A and C-K were analyzed by unpaired Student's *t*-test (*P < 0.05, **P < 0.01, ***P < 0.001, ****P < 0.0001 and NS, not significant).


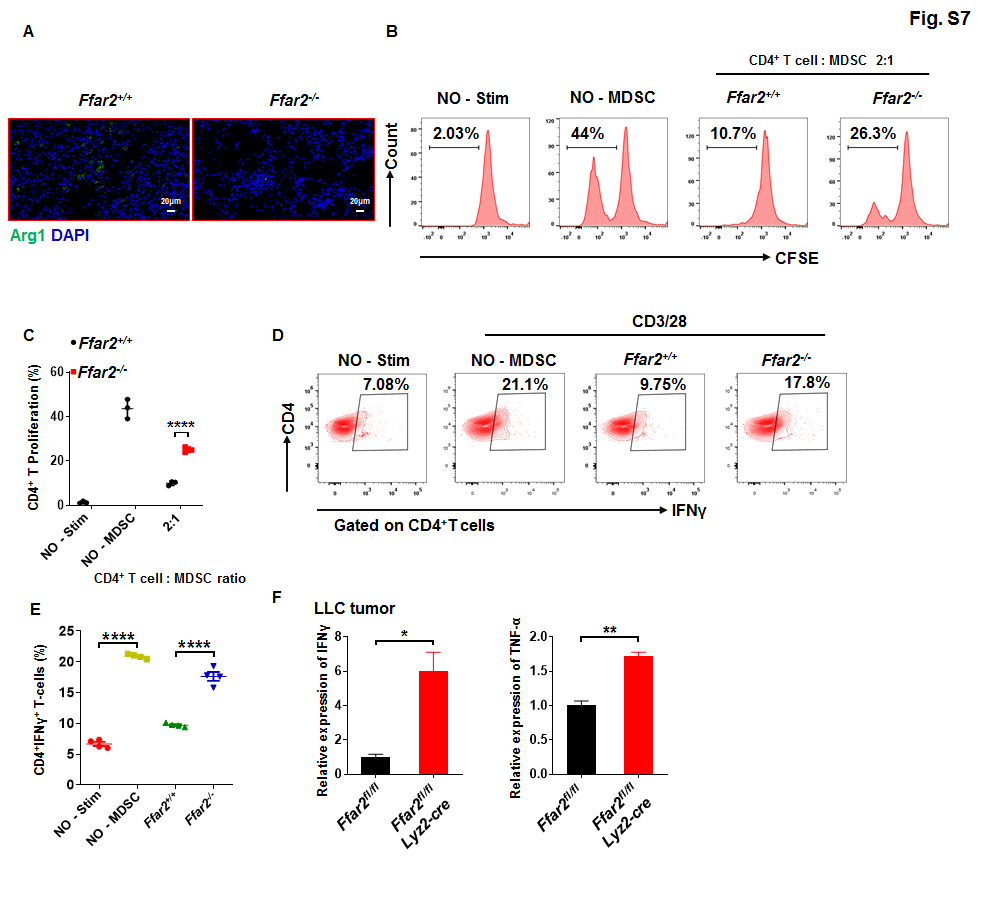


**Supplementary Figure 7.** **Reduced suppressive function in *Ffar2^-/-^* MDSCs.**

(A) Immunostaining analysis of Arg1 expression in urethane-induced *Ffar2^+/+^* and *Ffar2^-/-^* mice lung tumor nodules. B and C, suppression of CD4^+^ T cell proliferation in MDSCs isolated from *Ffar2^+/+^* or *Ffar2^-/-^* mice tumor-bearing mice spleen. MDSC-CD4^+^ T cell (CFSE-labelled T cells) suppression assay was analyzed by flow cytometry (B), and quantified (C) (n = 3). D and E, IFNγ expression of CD4^+^ T cell in co-cultured with MDSCs from *Ffar2^+/+^* or *Ffar2^-/-^* tumor-bearing mice spleen was analyzed by flow cytometry (D), and the percentage of CD4^+^IFNγ^+^ T-cells in total CD4^+^ T cells was quantified (E) (n = 4). (F) *Ffar2^fl/fl^ and Ffar2^fl/fl^Lyz2-cre* mice were injected subcutaneously with LLC cells (1 × 10^6^ cells/mouse). After 21 days of transplantation, single cell suspensions were prepared from tumors, and CD8^+^ T cells were isolated from the LLC tumors using mouse positive CD8^+^ T cells isolation kit. Relative mRNA levels of IFNγ and TNF-α in *Ffar2^fl/fl^* and *Ffar2^fl/fl^Lyz2-cre* LLC tumor-infiltration CD8^+^ T cell, which were determined by real-time RT-qPCR (n = 3). C, E and F are shown as mean ± SEM, and the experiment was performed three times and a representative example is shown. C, E and F were analyzed by unpaired Student's *t*-test (*P < 0.05, **P < 0.01, ***P < 0.001, ****P < 0.0001 and NS, not significant).


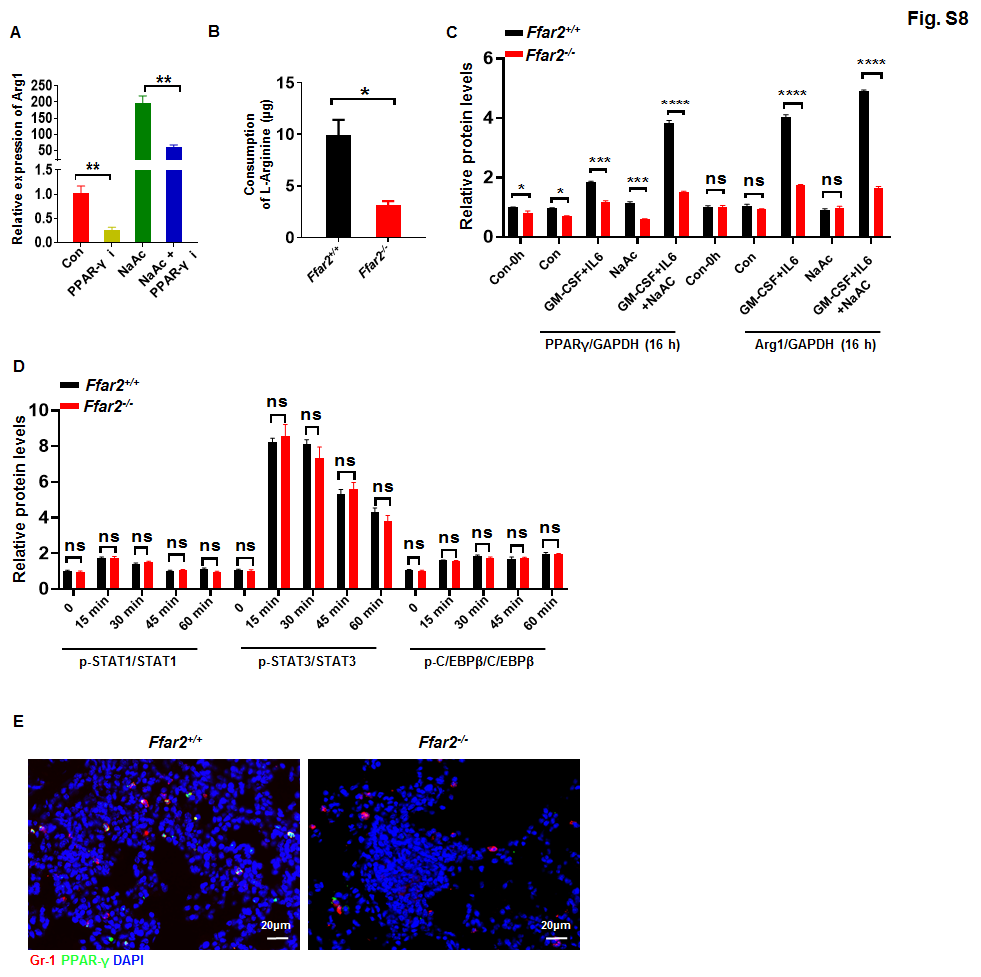


**Supplementary figure 8. FFAR2 deletion decrease the expression of Arg1 and consumption of L-Arginine in MDSCs in a PPAR-γ-dependent manner.**

(A) WT bone marrow-derived MDSCs (1 × 10^6^ cells/well) were seeded in a 6-well plate and cultured in complete RPMI 1640 medium overnight. After overnight resting, BM-MDSCs were pretreated with DMSO, PPAR-γ-inhibitor (GW9662; 2 μM), NaAc (5 mM) and PPAR-γ-inhibitor + NaAc for 2 hours before adding GM-CSF and IL-6, after 24 hours activated with GM-CSF (40 ng/ml) and IL-6 (40 ng/ml), relative mRNA levels of Arg1 was determined by real-time RT-qPCR (n = 3). (B) Consumption of L-Arginine by *Ffar2^+/+^* MDSCs and *Ffar2^-/-^* MDSCs (n = 3). (C) BM-MDSCs were activated by GM-CSF and IL6 with or without NaAc. The relative quantification expression of PPAR-γ and Arg1 were analyzed (n=3, biological replicates). (D) BM-MDSCs were activated by GM-CSF and IL6 for the indicated time. The relative quantification expression p-STAT1 (Tyr701), STAT1, p-STAT3 (Tyr705), STAT3, p-C/EBPβ (Thr217) and C/EBPβ were analyzed (n = 3, biological replicates). (E) Representative co-localization images of multicolor immunofluorescence staining for Gr-1 and PPAR-γ in urethane-induced lung tumor nodules. A-D are shown as mean ± SEM, and the experiment was performed three times and a representative example is shown. A-D were analyzed by unpaired Student's *t*-test (*P < 0.05, **P < 0.01, ***P < 0.001, ****P < 0.0001 and NS, not significant).


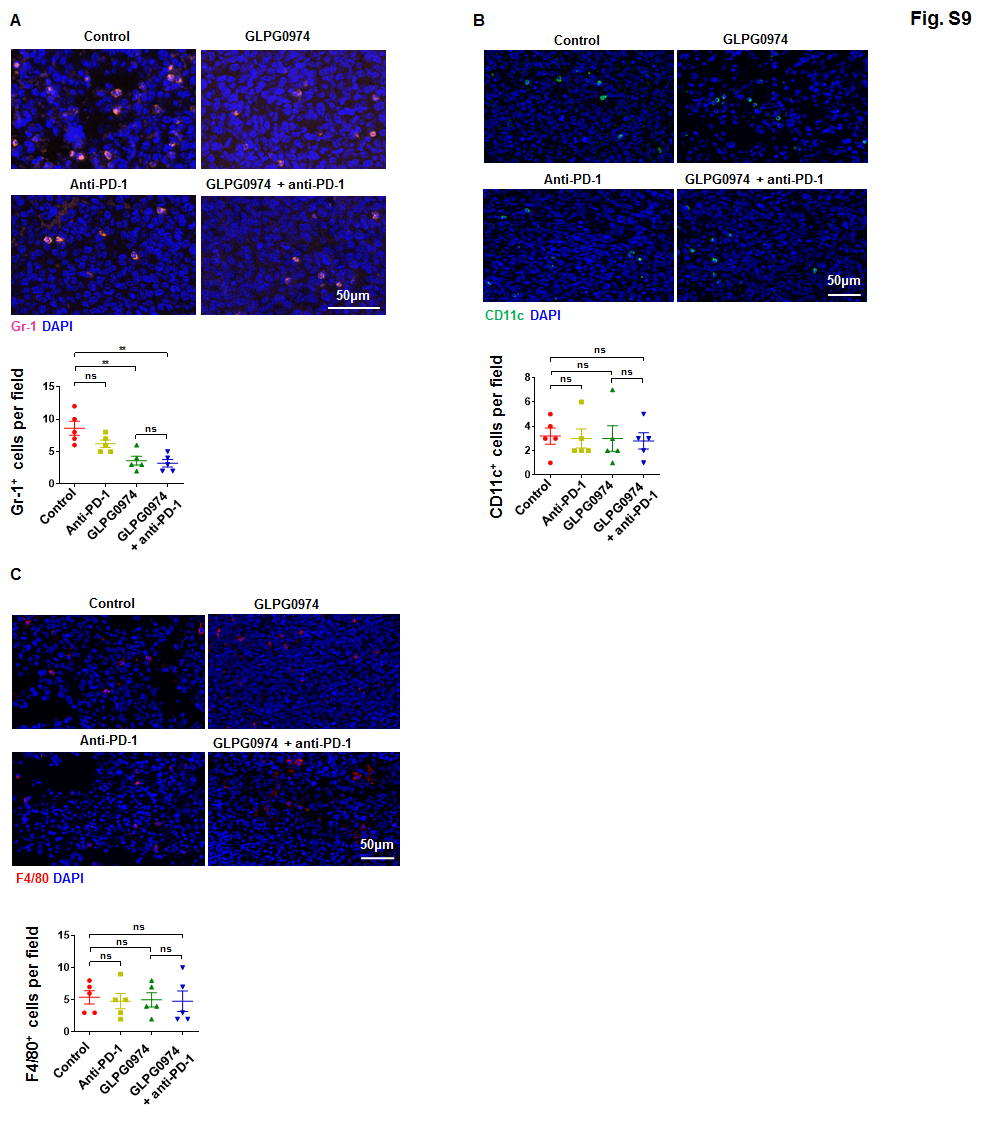


**Supplementary figure 9. The infiltration of MDSC, DC and macrophage in mouse LLC tumors upon different treatments.**

A-C, Representative images and quantification of immunofluorescence staining for Gr-1 (A) (n=5, biological replicates), CD11c (B) (n=5, biological replicates) and F4/80 (n=5, biological replicates) in mouse LLC tumors. Data are shown as mean ± SEM, and the experiment was performed three times and a representative example is shown. A-C were analyzed by one-way ANOVA (*P < 0.05, **P < 0.01, ***P < 0.001, ****P < 0.0001 and NS, not significant).

**References**

1. Qin J, Zhang G, Zhang X, Tan B, Lv Z, Liu M*, et al.* TLR-Activated Gap Junction Channels Protect Mice against Bacterial Infection through Extracellular UDP Release. J Immunol **2016**;196:1790-8
