# Supplementary material for: FFAR2 expressing myeloid-derived suppressor cells drive cancer immunoevasion
Source: J Hematol Oncol. 2024 Feb 24;17:9. doi: 10.1186/s13045-024-01529-6 (PMC10894476; doi:10.1186/s13045-024-01529-6)
Supplement: Supplementary file 2 — Additional file 2. Primers used for qPCR. [file 13045_2024_1529_MOESM2_ESM.docx]

Supplementary Data File 2 for:

**FFAR2 expressing myeloid-derived suppressor cells drive cancer immunoevasion**

**Authors:** Zeda Zhao^1#^, Juliang Qin^1#^, Ying Qian^1^, Chenshen Huang^2^, Xiaohong Liu^1^, Ning Wang^3^, Liqin Li^3^, Yuqing Chao^1^, Binghe Tan^4^, Na Zhang^4^, Min Qian^1^, Dali Li^1^, Mingyao Liu^1^, Bing Du^1^*

**Table S1. Primers used for qPCR**

| **Primer name** | **Sequence (5’–3’)** |
| --- | --- |
| Arg1-F | ATCGGAGCGCCTTTCTCAAAA |
| Arg1-R | GGTCTCTCACGTCATACTCTGTTTC |
| iNOS-F | TGTGGCTGTGCTCCATAGTT |
| iNOS-R | CTGGAGGGACCAGCCAAATC |
| IL12p40-F | GTGAAGCACCAAATTACTCCGG |
| IL12p40-R | GCTTCATCTGCAAGTTCTTGGG |
| TNFα-F | CATCTTGTCAAAATTCGAGTGACAA |
| TNFα-R | TGGGAGTAGACAAGGTACAACCC |
| IL10-F | GAAGACCCTCAGGATGCGG |
| IL10-R | ACCTGCTCCACTGCCTTGCT |
| FFAR2-F | GGCTTCTACAGCAGCATCTA |
| FFAR2-R | AAGCACACCAGGAAATTAAG |
| IFNγ-F | ATTGCGGGGTTGTATCTGGG |
| IFNγ-R | GGAAGCACCAGGTGTCAAGT |
| GAPDH-F | ACCCAGAAGACTGTGGATGG |
| GAPDH-R | TTCAGCTCAGGGATGACCTT |
